# Biofabrication of 3D-printed, pre-cross-linked alginate dialdehyde–gelatin (ADA–GEL) scaffolds for an *in vivo* metastatic arteriovenous loop tumor model

**DOI:** 10.3389/fbioe.2025.1657653

**Published:** 2025-11-03

**Authors:** Evelin Sandor, Tannaz Karimi, Rafael Schmid, Yvonne Kulicke, Stefanie Heltmann-Meyer, Carolin Eckert, Sonja K. Schmidt, Jonas Röder, Markus V. Heppt, Aldo R. Boccaccini, Raymund E. Horch, Anja K. Boßerhoff, Annika Kengelbach-Weigand, Andreas Arkudas

**Affiliations:** ^1^ Department of Plastic and Hand Surgery and Laboratory for Tissue Engineering and Regenerative Medicine, University Hospital Erlangen, Friedrich-Alexander University Erlangen-Nürnberg (FAU), Erlangen, Germany; ^2^ Institute of Biochemistry, Friedrich-Alexander University Erlangen-Nürnberg (FAU), Erlangen, Germany; ^3^ Institute of Biomaterials, Friedrich-Alexander University Erlangen-Nürnberg (FAU), Erlangen, Germany; ^4^ Department of Dermatology, Deutsches Zentrum Immuntherapie (DZI), CCC Erlangen-EMN, Bavarian Cancer Research Center (BZKF), University Hospital Erlangen, Friedrich-Alexander University Erlangen-Nürnberg (FAU), Erlangen, Germany; ^5^ Dermpath München, Laboratory for Dermatopathology, Oral Pathology and Molecular Pathology, Munich, Germany

**Keywords:** bioprinting, melanoma, vascularization, rat model, metastasis

## Abstract

Vascularized models mimicking tumor pathophysiology to investigate tumor characteristics are of high interest. The arteriovenous loop (AVL) model is an established method to vitalize bioengineered tissue grafts. In this model, an artificial vascular axis serves as the only connection between the living organism and the biomaterial. The objective of this study was to establish a three-dimensional (3D) printed, functional scaffold design for the AVL rodent model, in which human melanoma cells, derived from lymph node metastasis, are embedded in pre-cross-linked alginate dialdehyde-gelatin (ADA–GEL) and implanted in rats (N = 10) for 4 weeks. Bioink scaffolds were 3D-printed in two different shapes (n = 5), designed specifically for the AVL model’s isolation chamber. Before implantation, the swelling behavior of the biofabricates was analyzed *in vitro*. The biocompatibility of the pre-cross-linked ADA–GEL and the impact of the scaffold-morphology were examined through macroscopic analysis and immunohistological stainings. The fluid uptake ratio of the hydrogel resulted in size extension, a finding which is highly relevant for the AVL model’s closed system. Biofabricated scaffolds made of pre-cross-linked ADA–GEL remained stable *in vivo* and allowed for *de novo* fibrovascular tissue formation. The hypothesized biocompatibility of the analyzed hydrogel was confirmed. The two scaffold models exhibited differences regarding tumor growth and *de novo* fibrovascular tissue formation capacity. In both groups, metastatic cells were detected in the lymph nodes of rodents. The present study demonstrated that the AVL model is an excellent *in vivo* tool for melanoma research, combining biofabrication and vascularization with a high ability to replicate metastasis. At the same we conclude, that adapting the design of the biofabricated implants to the AVL model, depending specifically on the ink used, is of major importance.

## Introduction

Cutaneous melanoma is one of the most invasive forms of skin cancer. It arises from the malignant transformation of melanocytes (Rebecca, 2020) and is associated with a high mortality rate worldwide (Harries et al., 2016). Melanoma is characterized by rapid progression and early metastasis formation (Tas, 2012), which can occur even in primary tumors with low levels of penetration, primarily targeting the liver, lung, and lymph nodes (Schmid et al., 2024). Furthermore, melanoma cells are capable of entering a dormant stage during which they remain inactive and clinically undetectable for years in the human body (Singvogel and Schittek, 2024). These characteristics make human melanoma an excellent model for studying different stages of cancer developmental and metastatic properties.

Even though targeted therapies and immunotherapies have made significant progress in recent years, the effectiveness of these treatments remains limited due to the emergence of drug resistance, which often results in renewed disease progression (Jenkins and Fisher, 2021). Consequently, the treatment of melanoma remains a significant medical challenge. Similar to healthy tissues, the survival and growth of malignant tumors require the continuous supply of nutrients and the exchange of oxygen and metabolic waste (Lugano et al., 2020). Tumor angiogenesis, the process in which new vascular sprouts are formed through the dissemination of endothelial cells from host blood vessels to provide growing tissues with nutrients, has been identified as a hallmark of cancer and plays a crucial role in solid tumor development (Majidpoor and Mortezaee, 2021).

Although established monolayer cell culturing techniques have greatly contributed to our current understanding of cell biology, these models may not accurately reflect the complex structures of functional tissues found *in vivo* (Fontoura et al., 2020). Biofabrication, which is defined as the automated generation of functional healthy or cancerous tissues by combining living and non-living components, is an intensively evolving field of research and holds the potential to remodel *in vivo* like environments (Santoni et al., 2022). It uses three-dimensional printing (3D printing) techniques to precisely arrange cells within a natural or synthetic scaffold. These scaffolds are capable of holding living cells. This allows for the standardized variation of multiple parameters, such as cell density in 3D or the modification of the external cellular environment (Parrish et al., 2019). Consequently, biofabrication is frequently used to model the extracellular matrix in order to investigate cell-cell and cell-matrix interactions, and tumor cell behavior (Aazmi et al., 2024).

Being composed of hydrophilic biopolymers (Monavari et al., 2021), nature-derived hydrogels display a high water absorption capacity and therefore provide encapsulated cells with a highly hydrated milieu while also offering an optimal environment for cell attachment (Reakasame and Boccaccini, 2018). Hydrogels are therefore essential for the development of bioinks for biofabrication (Parrish et al., 2019; Khan et al., 2024; Jain et al., 2024). Alginate and gelatin are two non-synthetic, commercially available hydrogels, which are commonly used in biomedical approaches due to their biocompatible characteristics (Sarker et al., 2014a). Alginate is a polysaccharide originating from brown algae, showing similarities to the human extracellular matrix, and can be gelled rapidly (Gungor-Ozkerim et al., 2018). To improve its ability to enable cell adhesion and printability properties, alginate is often modified or blended with various biopolymers (Ege and Boccaccini, 2024). Gelatin is a biodegradable protein obtained through the acidic or basic hydrolysis of collagen, which is typically derived from porcine or bovine skin. It improves cellular activity by providing integrin-binding sequences (RGD) that facilitate cell attachment (Schipka et al., 2024). Alginate dialdehyde–gelatin (ADA–GEL) is a hydrogel formed through covalent bonds between a partially oxidized form of alginate (ADA) and gelatin (GEL). It can be structured three-dimensionally and gelled into scaffolds via ionic cross-linking (Sarker et al., 2014a; Ege and Boccaccini, 2024). ADA–GEL has been reported to be a suitable material for tissue engineering approaches and cellular studies (Dranseikiene et al., 2020). Additionally, ADA–GEL offers an optimal platform for melanoma cell proliferation (Schipka et al., 2024) and has shown compatibility with endothelial cells (Schulik et al., 2023), making this bioink highly suitable for studying melanoma behavior and tumor angiogenesis.

Vascularization of 3D-printed, tissue-like structures to establish a functional blood vessel network and thereby render the artificial tissue operational *in vivo* remains a well-recognized challenge in biofabrication (Datta et al., 2017). Several strategies to overcome this issue have been reported with increasing frequency (Paulsen and Miller, 2015; Ruther et al., 2018; Landau et al., 2024). One such strategy is the arteriovenous loop (AVL) model, which involves creating an artificial vascular axis by microsurgically anastomosing an artery, a vein, and an interpositional venous graft. The AVL is then transferred into an isolated implantation chamber, which can be filled with different bioinks and maintained in the animal for a defined period of time. The only contact between the animal and the implanted biofabricated tissue is the newly created vascular axis, only allowing intrinsic vascularization of the implanted scaffolds (Weigand et al., 2016). This model allows for the observation of tumor progression and tumor angiogenesis, among other phenomena, in a readily manipulable *in vivo* milieu (Schmid et al., 2024), and the assessment of angiogenic potential of hydrogels in living organisms (Heltmann-Meyer et al., 2021). This enables a more comprehensive evaluation of the effects of the 3D-printed systems on the vascular axis and vice versa.

The objective of this study was to develop a vascularized 3D-printed melanoma *in vivo* model. The model utilizes the physiological environment of the living organism as a bioreactor. In order to investigate melanoma tumor formation and progression, tumor cell-laden, pre-cross-linked ADA–GEL biofabricates were implanted in the AVL rat model using two different 3D bioprinting designs. These implantations were performed to examine the impact of different 3D printing strategies on the artificially generated, highly sensitive blood vessel network and the tumor formation capacity and metastatic activity of melanoma *in vivo*. Subsequently, the explanted constructs were analyzed histologically, and the two systems were compared. The results of this study may be valuable for the refinement and development of other 3D-printed *in vivo* models for tumor research and therapeutic development.

## Materials and methods

### Cell culture

For this study, a melanoma cell line derived from a human lymph node metastasis, Mel Im, was used. Cells were cultivated at 37 °C and 5% CO_2_ in Dulbecco’s modified Eagle’s medium (DMEM) low glucose (Sigma-Aldrich, St. Louis, MO, United States) with L-glutamine (2 mM, Sigma-Aldrich), 10% fetal bovine serum (FCS Superior, Sigma-Aldrich), and 1% penicillin/streptomycin (100 U mL^−1^, 0.1 mg mL^−1^, Sigma-Aldrich). The cell culture medium was changed three times per week.

### Biofabrication of implanted constructs

#### Preparation of pre-cross-linked ADA–GEL

Alginate dialdehyde (ADA)–gelatin (GEL) was prepared and cross-linked as described before (Hazur et al., 2020). ADA (6.25% (w/v)) with an oxidation degree of 13% and gelatin (6.25% (w/v)) were dissolved in Dulbecco’s phosphate-buffered saline (PBS, Sigma-Aldrich). After 30 min of stirring ADA at room temperature and gelatin at 37 °C, 250 mM CaCO_3_ was dispersed into the gelatin stock solution. Next, the ADA and GEL solutions were blended equally and stirred for 30 min at 37 °C. The mixture was then pre-cross-linked by adding dropwise 1 mL of 250 mM D-gluconolactone (GDL, Sigma-Aldrich) solution, which was dissolved in deionized water directly prior to use. The material was stirred for an additional 3 h at 37 °C and precooled for 4 min at 17 °C prior to 3D printing. 3D bioprinting and the cross-linking procedure were carried out at room temperature. Samples were not exposed to room temperature for more than 20 min, including during bioprinting and cross-linking.

In order to verify the successful synthesis of the ADA, Fourier-transform infrared spectroscopy (FT-IR) was carried out as described previously. In brief, attenuated total reflectance Fourier-transform infrared (ATR-FT-IR) spectroscopy (IRAffinity-1S, Shimadzu, Germany) analysis was performed in absorbance mode in the wavenumber range from 4,000 cm^−1^ to 400 cm^−1^ with a resolution of 4 cm^−1^ on ADA and pristine alginate (Sarker et al., 2014a). The materials were freeze-dried prior to ART-FT-IR analysis. The successful synthesis of ADA was confirmed (Supplementary Figure S1).

#### Implantation constructs

The implantation constructs were designed using Tinkercad (Autodesk©, San Francisco, CA, United States) and 3D-printed (CELLINK Inkredible+, CELLINK, Boston, MA, United States) using a sterile standard conical bioprinting nozzle 22G (CELLINK) at a pressure of 30–40 kPa and immersed for 10 min in 0.1 M CaCl_2_ solution containing 5% (w/v) microbial transglutaminase (mTG, Ajinomoto foods Europe, Paris, France) for cross-linking at room temperature. Afterward, samples were washed in the cell culture medium for 10 min at 37 °C. The constructs were then maintained at 37 °C in the corresponding medium and transferred to the surgery room. They were placed into the implantation chambers no later than 60 min after cross-linking.

### Group A

**FIGURE 1:**
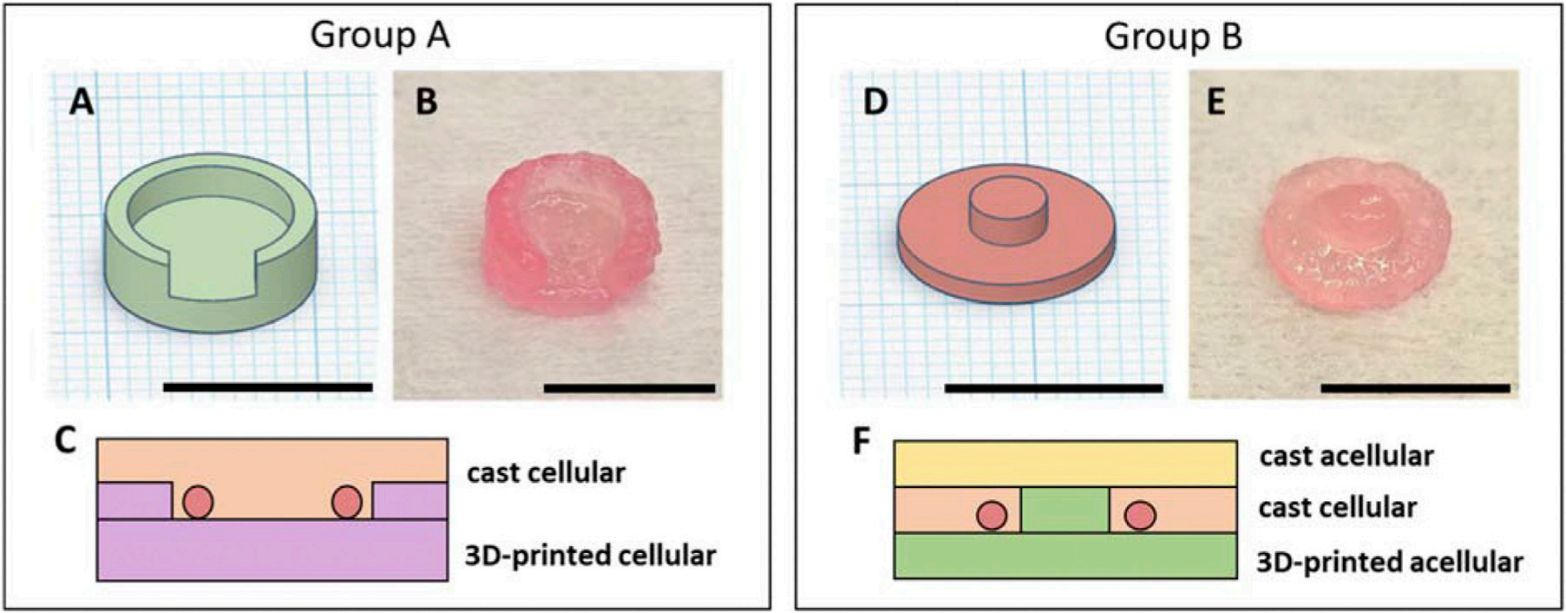
3D-printed bioink for AVL implantation: CAD models (designed using Tinkercad, Autodesk^©^) of implanted constructs **(A/D)**. 3D-printed implantation constructs using pre-cross-linked ADA–GEL after cross-linking and immersion in DMEM for 10 min **(B/E)**. Schematic cross-sections of the implantation constructs: the construct for group A consisted of a 3D-printed, cell-laden bottom part and a cast, cell containing upper part **(C)**; the construct for group B contained a 3D-printed acellular bottom part, a cellular cast middle layer, and an acellular cast upper layer **(F)**. Scale bars = 1 cm.

The implantation cellular construct for group A consisted of a disk-shaped structure with a diameter of 10 mm, connected to an outer wall with a height of 4.5 mm, and a 5 mm-long gap at the entrance of the chamber to accommodate the vessels (Figure 1A). A cell pellet of 1 × 10^6^ Mel Im cells was carefully resuspended in 900 µL pre-cross-linked ADA–GEL using a positive displacement pipette, and a printing cartridge (CELLINK) was loaded with the prepared bioink. Following the printing and cross-linking procedure (Figure 1B) of the hydrogel construct as described above, biofabricates were placed into the implantation chamber. Afterward, the arteriovenous loop (AVL) was placed onto the 3D-printed, cell-containing construct, and the remaining bioink volume was cast directly onto the vascular axis, completely filling the implantation chamber. It was then cross-linked *in situ* by pipetting dropwise 0.1 M CaCl_2_ containing 5% (w/v) mTG onto the construct (Figure 1C) for 10 min. Before the chambers were closed, constructs were washed by pipetting Hanks’ Balanced Salt solution (HBSS, Sigma-Aldrich).

### Group B

Acellular, pre-cross-linked ADA–GEL was printed in a two-part, cylinder-shaped form as a bottom portion for the implantation biofabricates of group B. The base segment had a diameter of 10 mm and a height of 1 mm, while the connecting midsection at the top measured a diameter of 3.5 mm and a height of 1.5 mm (Figures 1D,E). For cell implantation, a pellet of 1 × 10^6^ Mel Im cells was carefully resuspended in 100 µL pre-cross-linked ADA–GEL. After the AVL was placed onto the printed hydrogel part, the ADA–GEL (100 µL) with resuspended cells was directly cast onto the vascular axis, and the system was completed by casting an additional 100 µL of cell-free ADA–GEL and cross-linked *in situ* by pipetting dropwise 0.1 M CaCl_2_ containing 5% (w/v) mTG (Figure 1F) for 10 min. Before closing, constructs were washed with HBSS.

### Swelling assay of pre-cross-linked ADA–GEL

In order to determine the swelling properties of 3D-printed ADA–GEL implantation constructs, the weight and diameter of 3D-printed constructs of groups A and B were examined at different time points: 0, 1, 2, 3, 4, 18, and 24 h after cross-linking. Accordingly, both constructs were printed in 5 technical replicates and cross-linked as described below. Subsequently, the weight of each sample (W_0_) and the sample diameters (D_0_) were measured using a scale. Samples were maintained in the cell culture medium and incubated at 37 °C and 5% CO_2_. The weight and diameter percentage at different time points were obtained using the following formula:

$$W_{r}=\left(\frac{\text{Wt}}{W0}\right)\times 100.$$

Here, W_0_ represents the starting weight of the sample (immediately after cross-linking), and W_t_ represents the weight at specific time points.

$$D_{r}=\left(\frac{\text{Dt}}{D0}\right)\times 100.$$

Here, D_0_ represents the starting diameter of the sample (immediately after cross-linking), and D_t_ represents the diameter at specific time points.

### Animals

The AVL experiments involved 10 male Rowett nude rats (RNU, Crl: NIH-Foxn1rnu, Charles River Laboratories, Wilmington, MA, United States), divided randomly into group A (n = 5) and group B (n = 5), respectively. The animals, aged between 23–52 weeks, exhibited body weights ranging from 370 g to 470 g. Animals were anesthetized using isoflurane (CP-Pharma, Burgdorf, Germany) during the AVL surgery and received intravenous butorphanol (1.5 mg kg^−1^, CP-Pharma), Metacam (meloxicam, 2 mg kg^−1^, Boehringer Ingelheim Vetmedica GmbH, Ingelheim am Rhein, Germany), and enrofloxacin (7.5 mg kg^−1^, Bayer, Leverkusen, Germany) intraoperatively. Additionally, Metacam (2 mg kg^−1^) and enrofloxacin (7.5 mg kg^−1^) were administered subcutaneously for a 5-day time period, and enoxaparin sodium (10 mg kg^−1^, Clexane, Sanofi-Aventis Deutschland GmbH, Frankfurt am Main, Germany) was administered for a 2-day time period postoperatively. Each rat tolerated the surgical procedure and postoperative period well. All animal experiments were permitted by the animal care committee of the University of Erlangen-Nürnberg and the Government of Unterfranken, Germany (license number: 55.2.2-2532-2-1543-24).

### AVL surgery

**FIGURE 2:**
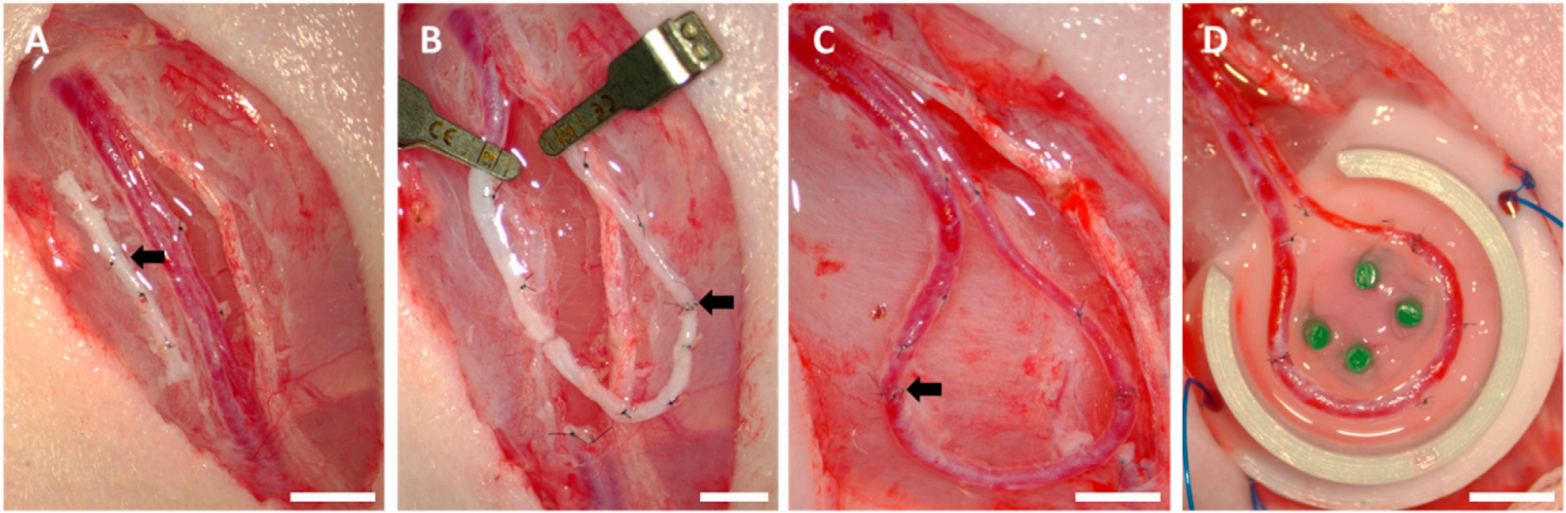
Arteriovenous loop surgery: **(A)** operation field with exposed vessels and interposed venous graft (black arrow), **(B)** first end-to-end anastomosis (black arrow) between the artery and venous graft, **(C)** second end-to-end anastomosis (black arrow) between the venous graft and vein, **(D)** positioning the AVL on the 3D-printed half of the implantation construct; scale bars = 5 mm.

The implantation surgeries were carried out as described previously (Schmid et al., 2024; Arkudas et al., 2013; Weigand et al., 2018) using an operative microscope (Carl Zeiss, Oberkochen, Germany) under sterile conditions. In summary, following the exposition of the left femoral vein and artery, a contralateral femoral venous graft was harvested (Figure 2A), and the adventitia was excised from each vascular end. Vessels were flushed using Ringer–heparin solution (100 IU mL^−1^, Ratiopharm GmbH, Ulm, Germany). Subsequently, the interpositional venous graft was anastomosed to the artery via its distal end (Figure 2B) and via its proximal end to the vein (Figure 2C). For the end-to-end anastomosis, single microsurgical knots were made with a non-resorbable 11–0 suture (Ethicon, Somerville, NJ, United States). Afterward, the 3D-printed hydrogel component was positioned in a polytetrafluoroethylene (PTFE) implantation chamber with an internal diameter of 10 mm and a wall height of 6 mm. Four plastic pins were inserted vertically through the chamber and gel to prevent displacement of the newly formed vascular axis. The AVL was then gently placed on the 3D-printed scaffold (Figure 2D). The isolation chamber was fixed to the limb musculature by means of single surgical knots with 6–0 sutures (Ethicon). Human fibrin clots (Baxter, Deerfield, IL, United States) were applied to close the chamber entrance and ensure a linear, minimal tensile position of the AVL at the entrance of the chamber. Thereafter, the chamber was filled with cell-laden hydrogel and closed with a round lid. The wound closure was accomplished by the use of subcutaneous (6–0, Ethicon) and simple skin sutures (4–0, Ethicon).

### Explantation procedure of AVL constructs

Animals were perfused after 4 weeks with yellow Microfil^®^ MV-122 contrast agent (Flow Tech Inc., Houston, TX, United States) as described previously (Osterloh et al., 2025). In brief, following a longitudinal laparotomy, the aorta and vena cava were surgically exposed. After the vena cava was punctured, the vascular network was flushed through the aorta using approximately 150 mL of Ringer–heparin solution (100 IU mL^−1^), then 8 mL Microfil^®^ MV-122 solution was mixed with 10 mL of diluent according to the manufacturer’s instructions, and prior to the injection into the aorta, 5% MV curing agent was added. All fluids used were preheated at 37 °C. Thereafter, animals were maintained at 4 °C for 24 h. Perfusion was carried out under general isoflurane anesthesia, and butorphanol (1.5 mg kg^−1^) was injected intravenously for analgesia. In addition to the biofabricates, lymph nodes (*Lymphonodus subiliacus*, lateral to the AVL, or *Lymphonodus lumbales*) were explanted and fixed overnight in 4% formaldehyde (Roti®-Histofix, Carl Roth GmbH & Co., KG, Karlsruhe, Germany) for further analysis.

### Histological analysis

The explanted AVL constructs were cut in the middle into a proximal and distal part, dehydrated, embedded in paraffin, and cut into 4 µm cross-sections perpendicular to the AVL’s longitudinal axis using a microtome (Leica Microsystems, Wetzlar, Germany). The explanted lymph nodes were prepared for the histological analysis, identical to the chamber explants, except for the cutting step. Standard hematoxylin and eosin (HE) and periodic acid–Schiff (PAS) stainings were performed in the Department of Pathology, University Hospital Erlangen, Germany, according to the standard protocols (Tur et al., 2025). Tissue growth areas were calculated manually on four HE-stained sections per chamber explant and expressed relative to the total area using ImageJ 1.52p (Schindelin et al., 2012). For the detection of melanoma cells in AVL chamber explants and rat lymph nodes, slides were stained with a mouse monoclonal anti-human HMB45 antibody (1:50, ENZ-C34930, Enzo Life Science, Farmingdale, NY, United States; in a final concentration of 0.4 µg mL^−1^, diluted with AB Diluent, ZytoVision, Bremerhaven, Germany) using the ZytoChem Plus (HRP) Polymer Staining Kit (Zytomed Systems, ZytoVision GmbH) according to the manufacturer’s instructions. HMB45-positive tissue areas were measured manually (ImageJ) using 2 HMB45-stained slides per chamber explant, and the tumor area was set in relation to the total specimen’s area for the AVL explants. To assess the histological results concerning the metastasis formation in rodent lymph node samples, the outcomes were classified into four groups. Category 0 comprises lymph nodes that display no HMB45-positive cells. Categories 1 and 2 included animals in which a few or a moderate number of melanoma cells were observed. Animals with a high number of melanoma cells in lymph nodes were classified as category 3. Proliferating cells were stained using a rabbit monoclonal antibody against Ki67 (1:200, clone SP6, RBK027-05, Zytomed Systems, diluted with AB Diluent) using the ZytoChem Plus (HRP) Polymer Staining Kit. Mesenchymal cells were stained with a mouse monoclonal antibody against vimentin (1:150, clone V9, MSK023, ZytoVision GmbH, diluted with AB Diluent) and the ZytoChem Plus (HRP) Polymer Staining Kit. To investigate the SOX10 protein, sections of formalin-fixed and paraffin-embedded tumor samples were stained using the anti-SOX-10 antibody (EP268, Cell Marque, Rocklin, CA, United States; RRID:AB 2941085) at a 1:100 dilution in Dako Real Antibody Diluent (Agilent Technologies, Santa Clara, CA, United States). Detection was performed following the alkaline phosphatase method using the ultraView Universal Alkaline Phosphatase Red Detection Kit and Hematoxylin II (both from Roche Diagnostics, Basel, Switzerland) for counterstaining of nuclei, according to the manufacturer’s instructions. SOX10 staining was performed in the Department of Dermatology, University Hospital Erlangen. For macrophage staining, slides were incubated with a mouse monoclonal anti rat CD68 antibody (MCA341R, Bio-Rad, Hercules, CA, United States) at a dilution of 1: 20 in AB Diluent, yielding a final concentration of 8 µg mL^−1^. CD68-positive macrophages were counted manually (ImageJ) on two samples per explants and expressed relative to the total area of the specimen. Anti-inflammatory macrophages were stained using a mouse monoclonal antibody against CD163 (1:300, clone ED2, MCA342GA, BioRad, diluted with AB Diluent, final concentration of 1.67 µg mL^−1^) and the Fast Red TR/Naphthol AS Kit (F4648-50SET, Sigma-Aldrich). Smooth muscle cells were visualized with a mouse monoclonal antibody against α-smooth muscle actin (α-SMA, 1:350, clone 1A4, MSK 030-05, ZytoVision GmbH, diluted with AB Diluent) and the Fast Red TR/Naphthol AS Kit according to the manufacturer’s instructions.

### Statistical analysis

Means ± standard deviations (SDs) or dot plots with means were used to illustrate the data. The Shapiro–Wilk normality test and the Mann–Whitney test were performed for statistical analysis using GraphPad Prism 10.2.0 (GraphPad Software, La Jolla, CA, United States). Experimental groups with a *p*-value of ≤ 0.05 were considered significantly different.

## Results

### Swelling behavior of pre-cross-linked ADA–GEL

**FIGURE 3:**
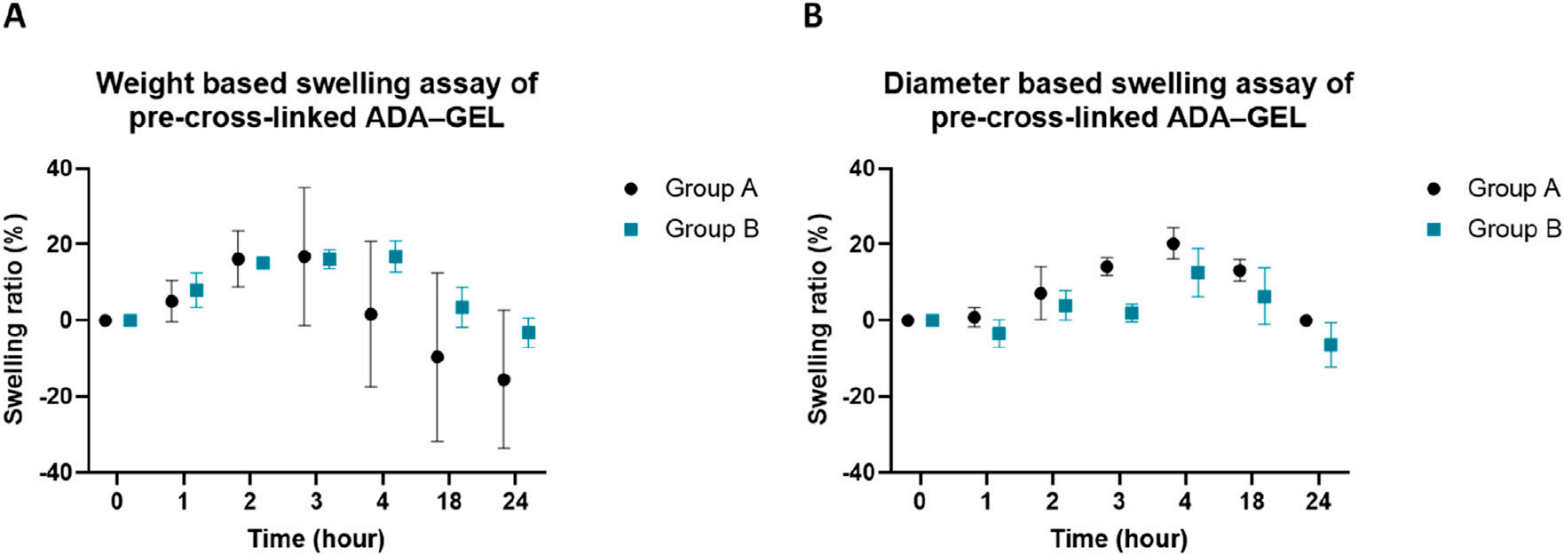
*In vitro* swelling assay of 3D-printed, pre-cross-linked ADA–GEL implantation scaffold constructs during 24 h; **(A)** calculation of the swelling ratio based on material weight extension of scaffold designs A and B (mean) and **(B)** calculation of the swelling ratio based on diameter extension of scaffold designs A and B (mean).

Because of their high capacity to absorb water, hydrogel scaffolds can extend their volume and therefore deform their shape in a damp environment. In this study, the 3D-printed hydrogels were transferred into a closed implantation chamber with defined dimensions. Therefore, swelling properties and shape deformations of implanted scaffolds can have an influence on the sensitive vascular systems. Focusing on establishing a functional 3D-printed AVL implantation system, the water absorption capacity of 3D-printed, pre-cross-linked ADA–GEL implantation constructs A and B was analyzed. Constructs of groups A and B were 3D-printed and maintained in the cell culture medium, changes in diameter and weight were recorded at certain time points. Within the first 4 h, scaffold deformations were macroscopically visible. According to the weight measurements (Figure 3A), the highest mean swelling rate compared to the start weight values was 16.80% (±18.13%) for construct A after 3 h. For construct B, the highest mean swelling rate of 16.80% (±4.01%) was measured after 4 h, compared to the original weight at the initial time point. Based on the changes in sample diameter (Figure 3B), the highest observed mean swelling rate for construct A was 20.26% (±4.01%) during the first 4 h, whereas for construct B, the highest mean value revealed 12.58% (±6.38%) after 4 h, compared to the initial diameter measurements. In both groups, constructs started to decrease in weight and diameter size after 4 h. After 24 h, the mean weight reduction compared to the initial values was calculated as 15.00% (±18.01%) for construct A, while for construct B, it was 3.18% (±3.92%). Interestingly, each sample from group A returned to the original diameter size, while the samples from group B exhibited a mean diameter reduction of 6.42% (±5.77%) after 24 h.

### *In vivo* biocompatibility and *de novo* tissue formation

**FIGURE 4:**
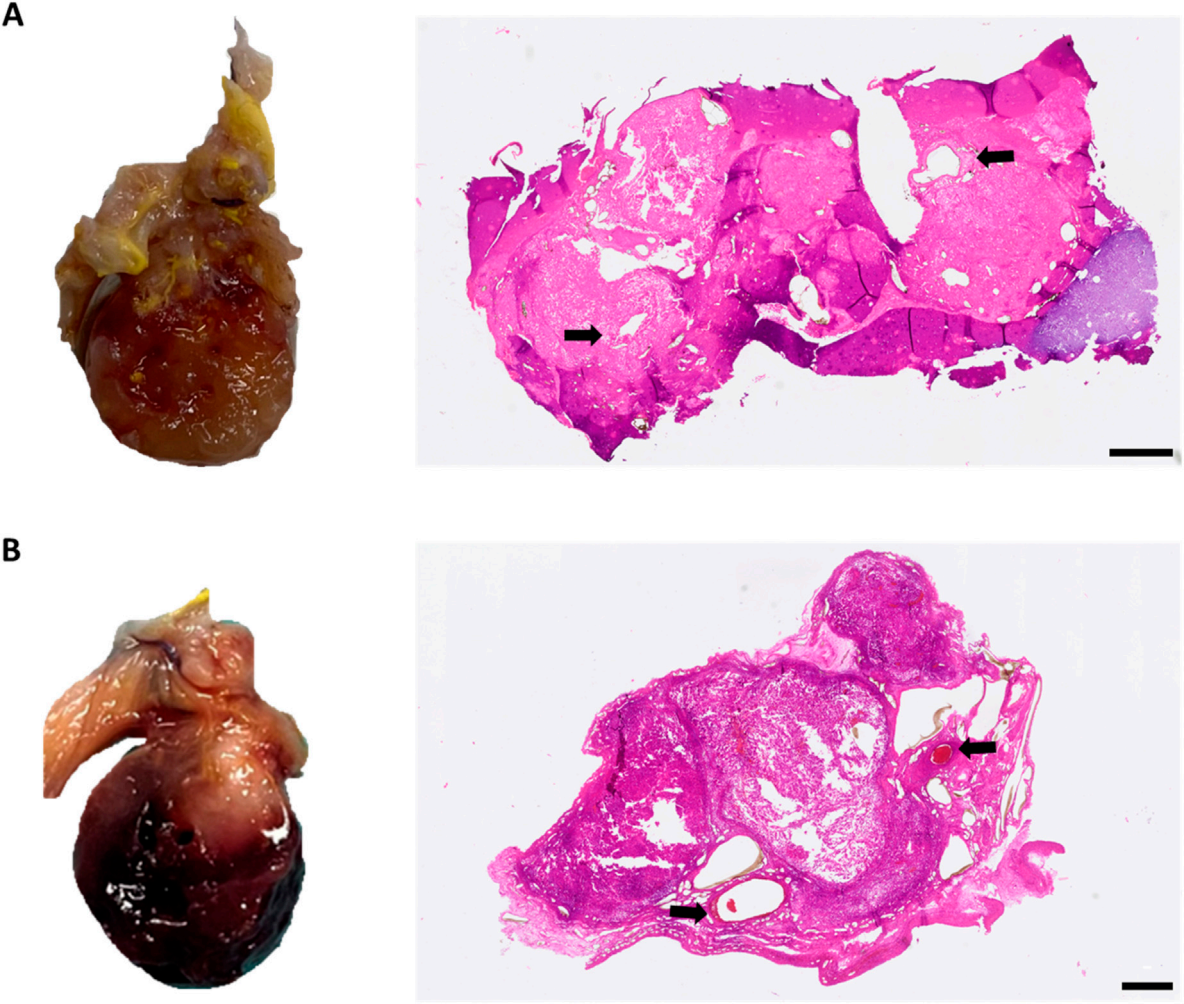
Macroscopic and microscopic appearance of tumor formation within the isolation chamber. (**(A)**, left) Macroscopic appearance of the explant with tumor growth in group A; (**(A)**, right) corresponding HE-stained histological cross-section. (**(B)**, left) Macroscopic appearance of the explant with tumor growth in group B; (**(B)** right) corresponding HE-stained histological cross-section. Black arrows indicate the host vasculature of the AVL; scale bars = 500 µm.

3D-printed, pre-cross-linked ADA–GEL constructs were implanted into the AVL rat model for 4 weeks, using two different scaffold shapes, specially designed for the AVL model’s isolation chamber. All rats survived and tolerated both the surgical procedures and the postoperative period without complications. No complications such as chamber dislocation, infections, or wound healing disorders occurred. The constructs were explanted 4 weeks post-implantation, and histological examinations were performed to study the presence of neovascularization, the tissue and tumor ingrowth regions, and immunological compatibility. At the time of explantation, explants from groups A and B could be easily removed from chambers and the hydrogel constructs appeared macroscopically in stable form (Figures 4A,B). Hydrogels showed no signs of significant degradation or resorption. Interestingly, explants with tumor growth appeared darkly discolored (Figures 4A,B), whereas explants from animals without tumor growth showed a light white-yellow color, regardless of the experimental group. Quantifying the cross-sectional areas did not show any significant variability between the two groups. The cross-sectional area averaged 8.643 mm^2^ (±3.167 mm^2^) for group A and 8.493 mm^2^ (±5.463 mm^2^) for group B. Additionally, in all animals, fibrovascular connective tissue grew around the implantation chambers, and fibrin gel was replaced by highly vascularized tissue masses at the chamber entrances. The vessels of newly formed connective tissue at the chamber entrances were perfused with yellow Microfil^®^, and additional yellow, presumably newly formed vessels were observed throughout the explanted gel constructs.

**FIGURE 5:**
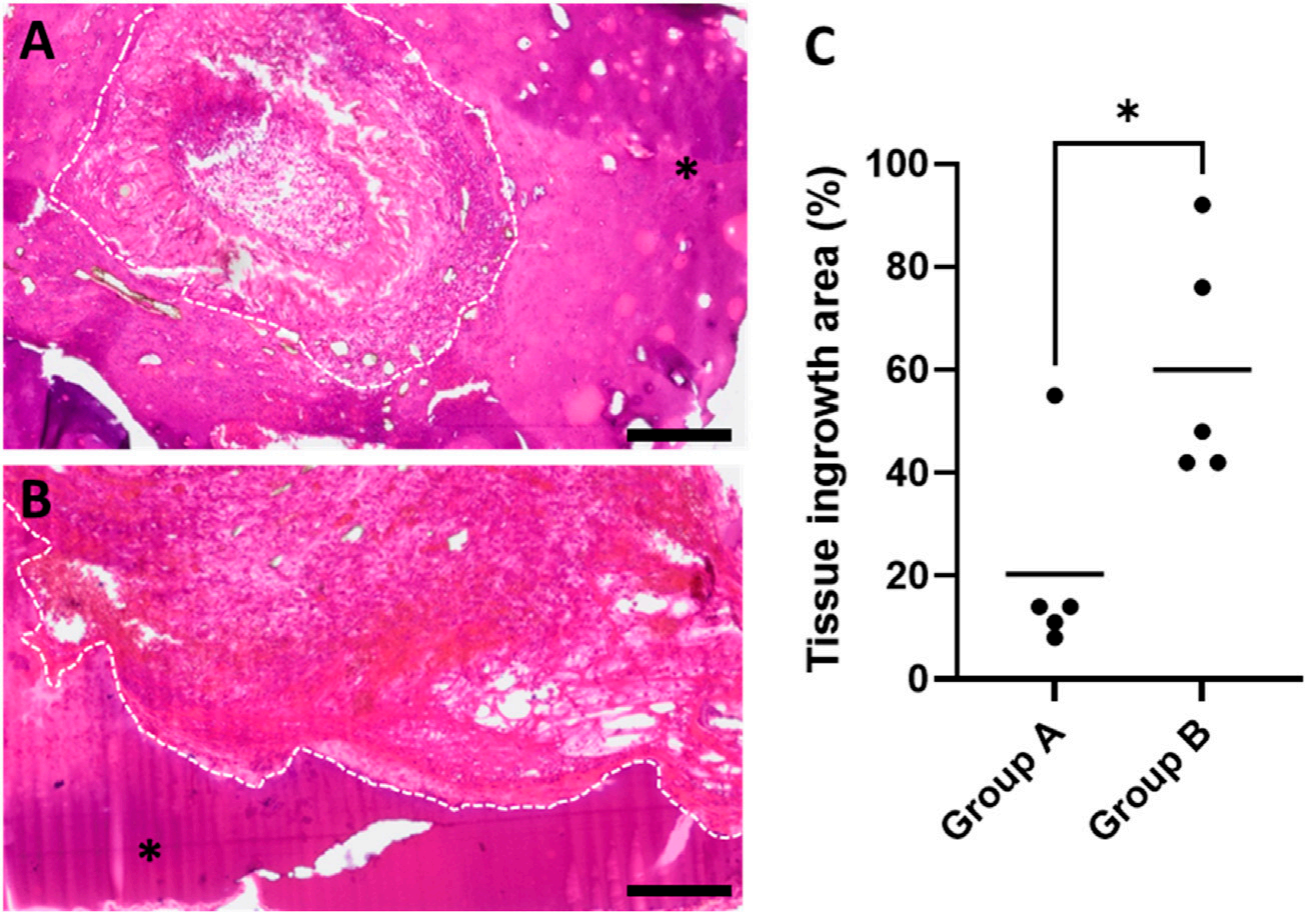
HE-stained histological cross-sections of exemplary animals highlighting newly formed connective tissue areas for **(A)** group A and **(B)** group B; asterisks mark the remaining hydrogel, and dashed white lines frame the margins between newly formed tissue areas and hydrogel; scale bars = 200 μm. **(C)** Quantification of tissue ingrowth areas revealed a significant difference between constructs A and B (with the mean of four sections of biological replicates, n = 5) (*p ≤ 0.05, Mann–Whitney test).

To examine whether ADA–GEL and the different construct designs can promote new tissue formation, HE-stained cross-sections were used to evaluate the ingrown tissue areas within the explants (Figures 5A,B). A statistically significant difference was observed between groups A and B. The ingrowth area averaged 20.42% (±19.47%) and 60.02% (±22.81%) of the scaffold area for groups A and B, respectively (Figure 5C). Tissue areas were predominantly located around the major vessels and spread out by replacing the hydrogel. Furthermore, the remaining ADA–GEL was also visually present in explants of groups A and B.

### Tumor growth

**FIGURE 6:**
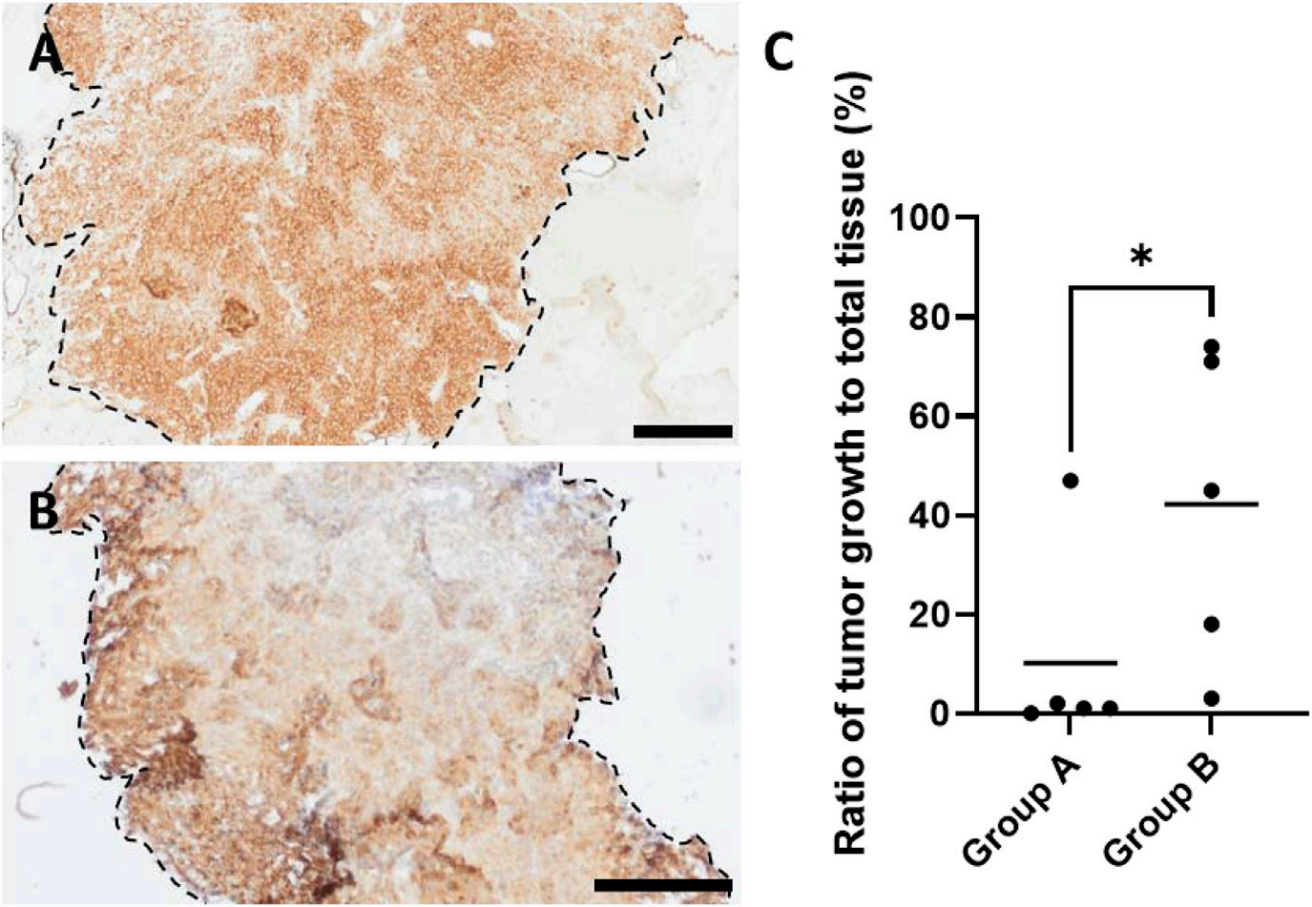
HMB45-stained histological cross-sections of exemplary animals of **(A)** group A and **(B)** group B highlighting HMB45-positive tumor areas (brown color indicates HMB45-positive melanoma cells), where dashed black lines frame the margins between tumor areas and hydrogel, and asterisks mark the remaining hydrogel; scale bars = 200 μm. **(C)** Quantification of tumor growth areas in relation to total tissue showed significant differences between groups A and B (with the mean of 2 sections of biological replicates, n = 5) (*p ≤ 0.05, Mann–Whitney test).

Tumor area quantification in HMB45-stained histological cross-sections of the chamber explants differed significantly between groups A and B (Figure 6). The mean tumor area to the total specimen area ratio revealed 10.00% (with a minimum value of 0.67% and a maximum value of 46.85%) in group A. One out of five animals in group A displayed a significant tumor growth with a ratio of tumor to total sample area of 46.85% (±0.005%). In the other four animals, HMB45-positive cells were observed mainly in single cell form, distributed within the explanted constructs, and clustered tumor cells were barely observable, resulting in a mean value of 0.785% (±0.007%). In contrast, group B’s mean tumor area was calculated to be 42.46% (±31.47%), with 2 out of 5 animals exhibiting a tumor growth area exceeding 70%. In one explant of group B, only single HMB45-positive cells were visible; however, in every other sample, tumor cell-clusters or growing tumor areas were observed. Without a preference for artery or vein, tumors grew primarily around the main vessels. Irrespective of the experimental group, HMB45-positive single cells and clusters were observed in the peripheral regions of the chambers that were not connected to the main tumor masses, exceeding 15% of the tumor growth area. Tumors were predominantly located in the proximal half of the explants, closer to the chamber entrance.

**FIGURE 7:**
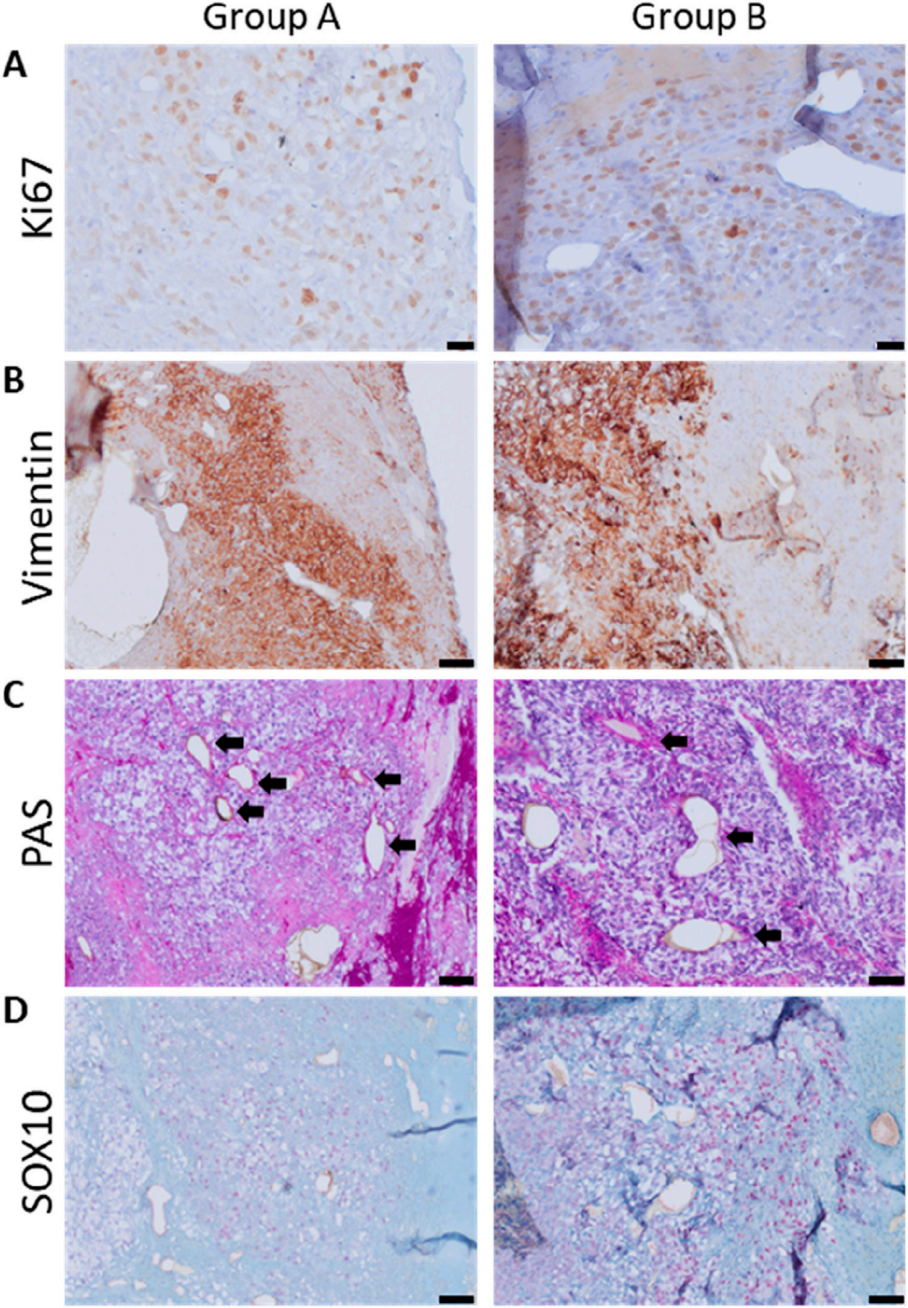
Immunohistological visualization of the tumor microenvironment; **(A)** proliferation: Ki67-positive cells were brown-stained; scale bars = 20 μm. **(B)** Mesenchymal cells positive for vimentin are brown-stained; scale bars = 50 μm. **(C)** PAS histology of grown tumors; arrows indicate capillaries; scale bars = 50 µm. **(D)** Heterogeneous expression of SOX10, a melanoma plasticity marker, in tumor masses; scale bar = 50 μm (left: group A and right: group B).

Ki67 histology was conducted to visualize tumor cell proliferation and allow for a deeper examination of the melanoma tumor environment. Ki67-positive cells were observed in a low number within tumor growth areas (Figure 7A) and showed a tendency to be found near the host vessels and at the periphery of the tumor formation regions, where the two experimental groups showed no differences. Vimentin-positive mesenchymal cells were abundant within the cancerous areas (Figure 7B). Additionally, single mesenchymal cells, possibly of rat origin, were observed in the connective tissue regions around the tumor masses. Differences between the two groups with regard to the location or the number of vimentin-positive mesenchymal cells inside the tumor growth areas were not observed. The use of PAS histology enabled the observation of micro-capillaries. The melanoma tumor areas contained numerous micro-capillaries (Figure 7C), regardless of the implanted scaffold’s shape, and without a detectable locational preference. Moreover, PAS-positive networks were observed in cancerous tissue growth. Immunohistochemistry for the melanoma plasticity marker SOX10 showed an inhomogeneous expression in the detected tumor tissue (Figure 7D).

### Immune response

**FIGURE 8:**
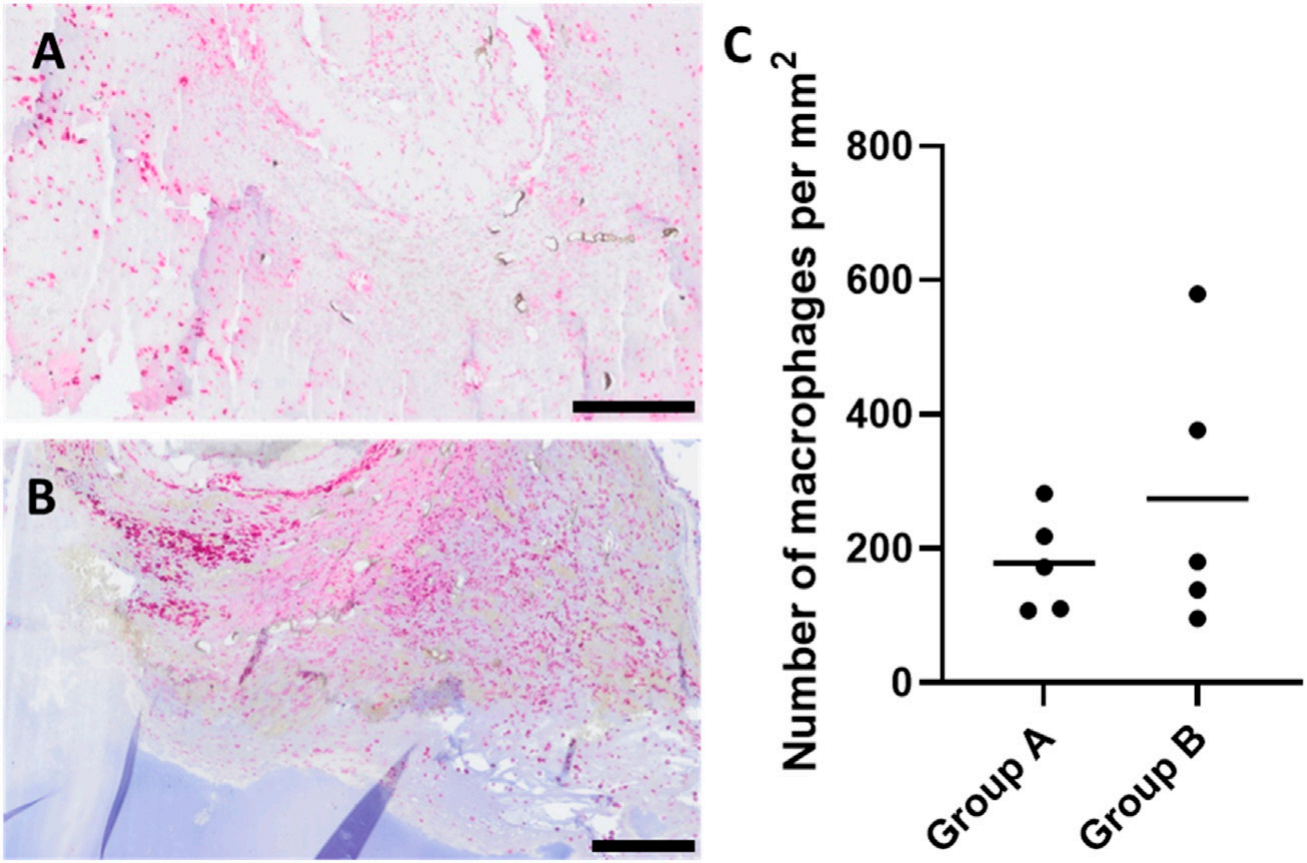
CD68-stained histological cross-sections of representative animals showing macrophage-infiltrated areas of chamber explants (red color indicates CD68-positive cells) **(A)** group A and **(B)** group B; scale bars = 200 μm. **(C)** Quantification of CD68-positive immune cells per mm^2^ did not differ significantly between the two experimental groups (with the mean of 2 sections of biological replicates, n = 5).

To evaluate the immune response and assess the biocompatibility of ADA–GEL, anti-CD68 staining was carried out (Figures 8A,B). CD68-positive multinuclear giant cells, as a sign of severe immune response, were not detectable. Only single, mononuclear macrophages were detected in both experimental groups. The quantity of CD68-positive cells did not differ significantly in group A 178.22 (±74.33 mm^−2^) and group B 274.01 (±201.70 mm^−2^) (Figure 8C). Macrophages tended to be mainly located surrounding the AVL; moreover, newly formed tissue areas were also abundantly infiltrated. Furthermore, macrophages were found to infiltrate tumor areas, with a tendency to be more prevalent at the hydrogel and tumor margins. Notably, a few macrophages were observed in ADA–GEL without direct contact with growing tissues.

**FIGURE 9:**
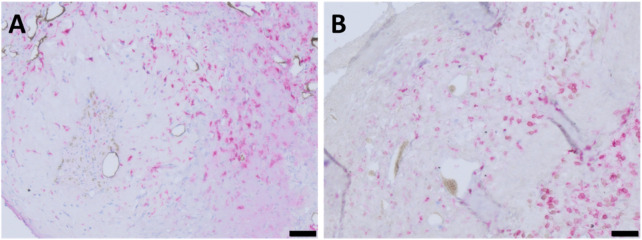
CD163-stained histological cross-sections of representative animals highlighting M2 macrophage-infiltrated areas of chamber explants (red color indicates CD163-positive cells) **(A)** group A and **(B)** group B; scale bars = 50 µm.

To provide deeper insights into immune cell infiltration, CD163 histology was carried out in order to be able to detect anti-inflammatory (M2) macrophages. *De novo*-formed connective tissues were found to be richly infiltrated by CD163-positive macrophages (Figure 9). Although single M2 cells were observed around the host vessels and at the peripheries of cancerous tissue masses, a considerable infiltration of M2 macrophages was not noted within the tumors. Our observations did not differ between the two groups with regard to CD163 staining.

### Vascularization

**FIGURE 10:**
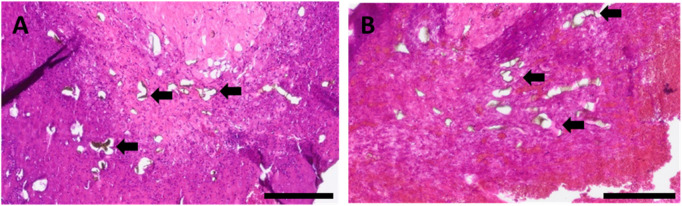
HE-stained histological cross-sections of representative animals of **(A)** group A and **(B)** group B, highlighting newly formed capillaries filled with Microfil^®^ (black arrows); scale bars = 200 µm.

Perfusion with yellow Microfil^®^ enabled visualization of the vascularization within the chamber explants as the intravascularly located Microfil^®^ appears black in histological sections. Formation of new vessels was observed in newly formed connective tissue masses in both experimental groups originating from the AVL (Figure 10). Moreover, Microfil^®^-filled capillaries were visible both within and adjacent to the tumor growth areas. Additionally, neovascularization in gel areas lacking tissue growth was not detectable. Notably, remarkable deformation of the vessel lumens in the main vascular axis, which appeared to impede blood flow through the biofabricates, was observed in both groups.

**FIGURE 11:**
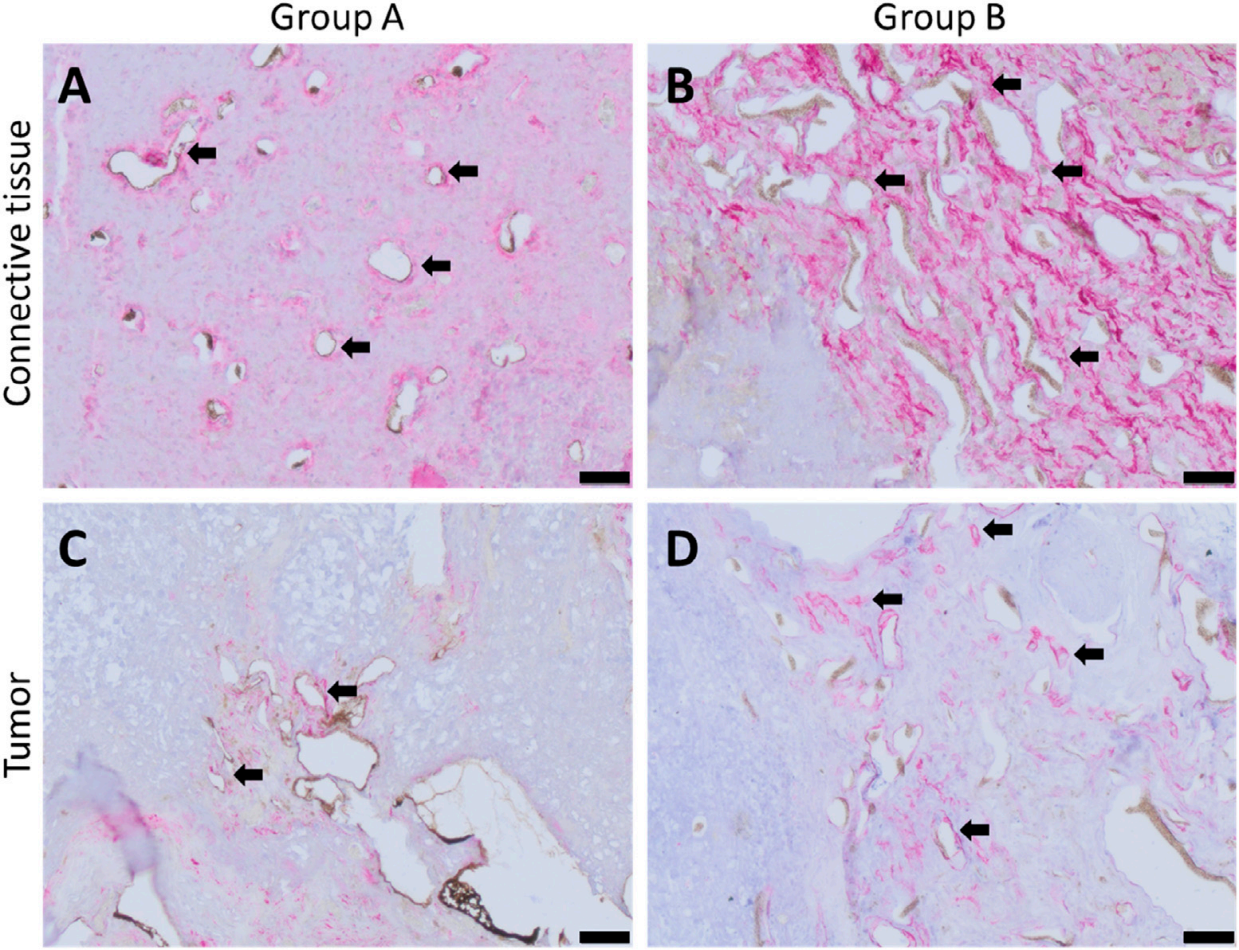
α-smooth muscle actin-stained histological cross-sections of represented animals showing red-stained smooth muscle cells; **(A)** *de novo*-formed fibrovascular tissue area for group A, **(B)** *de novo*-formed fibrovascular tissue area for group B, **(C)** vascularized tumorous tissue for group A, and **(D)** vascularized tumorous tissue for group B; black arrows point exemplary blood vessels. Scale bars = 50 µm.

Neovascularization was further visualized by immunostaining with an anti-α-smooth muscle actin antibody. In agreement with Microfil^®^ results, the newly developed fibrovascular tissue masses and tumor growth areas were found to be highly vascularized (Figure 11), where the neo-vessel formation tended to emerge from the host vessels, without dissimilarity between the two groups.

### Metastasis formation

**FIGURE 12:**
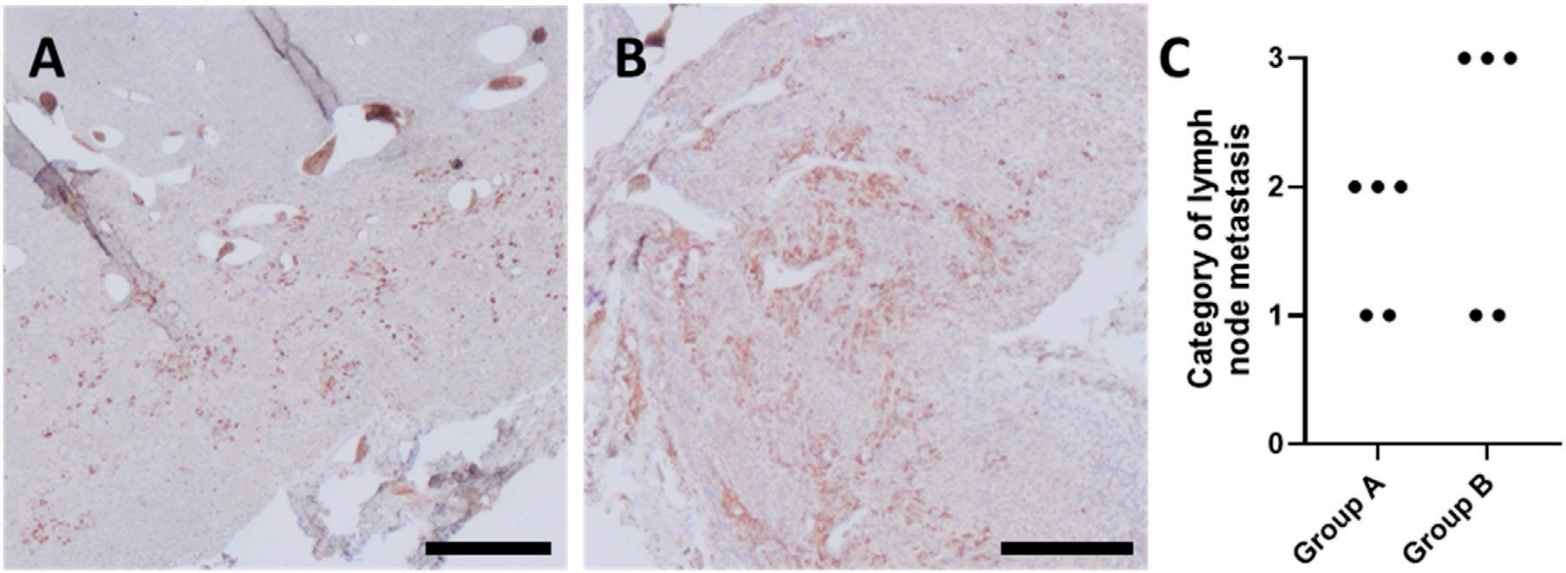
HMB45-stained histological cross-sections of representative animals of **(A)** group A and **(B)** group B, highlighting tissue areas of explanted lymph nodes infiltrated with metastatic melanoma cells (brown stained cells); scale bars = 200 μm. **(C)** Categorization of lymph node metastasis based on the presence of HMB45-positive cells, where category 0: no, category 1: few, category 2: moderate, and category 3: an abundant number of HMB45-positive cells were observed in explanted lymph nodes.

In order to observe the metastatic activity of implanted tumor cells and evaluate the AVL model’s functionality as a melanoma metastasis model using the different scaffold shapes, efferent lymph nodes (*Lymphonodus subiliacus*, occurring lateral to the AVL, or *Lymphonodus lumbales*) were explanted and stained for melanoma cells. HMB45-positive cells were observed in each lymph node explant in both experimental groups, irrespective of the tumor growth level (Figures 12A,B). In group A, metastatic cells were predominantly observed as single cells or duplets, with a low number of melanoma clusters detected in each animal, except one, in which metastatic cells were detected only as single cells or duplets. In contrast to group A, in lymph node explants of group B, in addition to metastatic single cells, cells were predominantly found in clusters. To assess the histological results concerning the metastasis formation, the results were classified into four groups based on the presence level of detected HMB45-positive cells. Since metastatic cells were observed in each animal (Supplementary Figure S2), none were categorized into group 0. For group A, two animals were classified in category 1 and three in category 2, whereas for group B of scaffold shape, three animals were assessed in category 3 and two in category 1 (Figure 12C).

## Discussion

Despite the progress of several bioengineering strategies concentrating on the *in vitro* generation of a functional vascular network, integrating a perfusable vasculature in order to vitalize bioengineered tissue grafts remains one of the leading challenges in the field of biofabrication (Datta et al., 2017). *In vivo* techniques, which utilize pre-existing vascular systems and employ the living organism as a bioreactor, offer an additional approach to address this issue (Weigand et al., 2018). The AVL system represents an *in vivo* strategy to induce intrinsic vascularization and *de novo* tissue generation (Zhan et al., 2016) in tissue-like biofabricates. The AVL rat model is based on a microsurgically anastomosed host vascular axis comprising a vein, an artery, and an interposed venous graft (Arkudas et al., 2013), which can subsequently be embedded into a well-defined implantation chamber (Weigand et al., 2016) filled with various cellular or acellular scaffold matrices (Zhan et al., 2016). In addition to being an optimally suited model for evaluating scaffold materials in terms of vascularization capacity and biocompatibility for tissue engineering approaches (Steiner et al., 2018), the transfer of the AVL model into a tumor model has been effectively used as a platform for *in vivo* cancer research. Compared to other *in vivo* tumor models, such as models of injecting tumor cells intravenously or subcutaneously, the AVL isolation chamber offers the advantage of excluding the impacts of the surrounding tissue regions, allowing for the creation of a well-characterized and easy-to-manipulate micromilieu (Weigand et al., 2018), and has a higher angiogenic potential compared to the so-called AV bundle model (Arkudas et al., 2013), which is based on the distal ligation of an artery and its associated vein (Polykandriotis et al., 2007).

As previously demonstrated, the AVL system combined with various cast hydrogels is a beneficial tool for studying malignant melanoma *in vivo* (Schmid et al., 2022). Furthermore, it has also been reported that various bioink characteristics can influence melanoma cell behavior differently and cause a significant variation in primary tumor growth using the AVL system, thereby making it possible to induce progressive and dormant tumor development stages of malignant melanoma *in vivo* (Schmid et al., 2024).

In the current study, we evaluated the effects of two different 3D-printed pre-cross-linked ADA–GEL designs on the highly sensitive AVL rat model with regard to the system’s functionality as a melanoma metastatic model. ADA–GEL, produced by covalent bonding of oxidized alginate and gelatin, displays many interesting features for *in vivo* use (Rottensteiner et al., 2014), including non-toxicity and proangiogenic characteristics (Steiner et al., 2018). In order to improve its printability properties and shape fidelity after the 3D printing process, introducing a pre-cross-linking step to the preparation process of ADA–GEL, as used in this study, was found to be beneficial (Hazur et al., 2020). Moreover, in addition to high melanoma (Schipka et al., 2024) and endothelial cell survival rate (Schulik et al., 2023), proliferative cell behavior and migration of melanoma cells could be detected in pre-cross-linked ADA–GEL in previous *in vitro* studies (Schipka et al., 2024). Taken together, these properties make pre-cross-linked ADA–GEL an excellent candidate for the establishment of a metastatic melanoma model in fusion with the AVL system.

Swelling characteristics are an important consideration when selecting bioactive hydrogels for biomedical applications. For instance, shrinkable hydrogels can attach greatly to the skin and exert contractile forces on lesions, thereby supporting wound contraction, while the application of highly swelling hydrogels in the human internal soft-tissue environment can lead to medical complications. Concurrently, factors that influence swelling, such as pore size or intermolecular spaces within the hydrogels (Feng and Wang, 2023), are crucial when incorporating living cells into hydrogels and need to be taken into consideration simultaneously with water absorption capacity, according to the application goal. Nonetheless, the ability to absorb, retain, and diffuse biological solutions is one of the most important characteristics of hydrogels (Sarker et al., 2014a). On the other hand, water absorption can apparently influence the shape of implanted scaffolds. Since the 3D-printed hydrogels in the AVL model are transferred into a sealed isolation chamber with defined dimensions, swelling properties leading to volumetric expansion and deformation of scaffolds can have a negative effect on the sensitive vasculature. As a result of water absorption-induced size extension, hydrogel scaffolds may exert compression on vessels, potentially leading to lumen deformation and thrombus formation. Nevertheless, it must be mentioned that volumetric extension rates of scaffolds tested in *in vitro* conditions do not necessarily correspond to the *in vivo* conditions. However, a high amount of biological fluids in contact with the hydrogel is applied during an AVL surgery and can serve as a water source. To minimize technical issues arising from material expansion in the AVL model, when a highly swelling hydrogel is selected, it is advantageous to evaluate the swelling behavior *in vitro* prior to designing the implantation scaffold. Moreover, leaving the implantation chamber partially unfilled with hydrogel, thereby allowing space for scaffold expansion—as was done with ADA–GEL group B of the current study—was found to be advantageous. It is also recommended to remove fluids that may come into contact with the hydrogel constructs such as blood, cross-linking agents, or flushing solutions, before closing the chamber system intraoperatively.

Previous studies on the swelling behavior of non-pre-cross-linked ADA–GEL demonstrated a rapid water intake within the first 3 h after cross-linking and a high degree of degradation during long-term *in vitro* incubation (Bider et al., 2024). Pre-cross-linking ADA–GEL with CaCl_2_ as a source of Ca^2+^ ions resulted in a lower swelling rate, leading to a 20%–30% water uptake ratio within the first 6 h after cross-linking (Malandrino et al., 2024). This is consistent with our measurements of pre-cross-linked ADA–GEL constructs, where CaCO_3_ was used for pre-cross-linking. The increase in weight and diameter of both construct designs reached their peak within the first 4 h, and the swelling ratios did not differ between the used scaffold designs noticeably (Figure 3). The decreased swelling tendency of pre-cross-linked ADA–GEL observed after 3 and 4 h can be attributed, on one hand, to hydrogel saturation with water and, on the other hand, to the onset of material degradation (Sarker et al., 2014b). Cells have the potential to grow more intensively in the degraded areas, which simultaneously allows for *de novo* tissue formation. Pre-cross-linked ADA–GEL has been found to display favorable stability and degradation characteristics for tumor modeling (Schipka et al., 2024). The chamber systems did not collapse due to material degradation since the degrading hydrogel was replaced by freshly growing tissue masses. This also indicates a good biocompatibility of pre-cross-linked ADA–GEL. The mechanical stiffness of hydrogels is another important property of biomaterials, which can influence container tissue systems. The currently used composition of ADA–GEL was confirmed to possess a mechanical stiffness comparable to human soft tissue (Schulik et al., 2023). Consequently, pre-cross-linked ADA–GEL can support cell survival, spreading, and migration (Schipka et al., 2024). The ability of cells to migrate through the hydrogel matrix and reach the vascular network plays a crucial role when establishing metastatic cancer AVL models. Because the implanted material has a similar mechanical stiffness to the physiological soft tissue, we do not assume that material stiffness has a negative effect on the AVL. Focusing on how to improve pre-existing AVL systems, extrusion-based 3D printing of implanted hydrogel scaffolds as a novel component was introduced. Computer-controlled 3D printing offers the advantage of precise 3D cell arrangement and allows for a more precise definition of the cellular composition of the tumor micromilieu. This increases the model’s complexity and its ability for further calibration (Shukla et al., 2022) compared to cast hydrogel implantation systems. The scaffold constructs were designed to have the same diameter as the inner diameter of the isolation chamber. Construct A was twice as high as construct B and had an outer wall, encapsulating the vascular axis outwardly (Figure 1). Construct B was designed without an outer wall, allowing more space for the AVL. The volume of implanted hydrogel for construct B was calculated so as not to completely fill the chamber; hence, more space was available for swelling-induced material expansion, preventing compression of the vessels. In contrast, construct A filled nearly the entire chamber in its non-swollen state, leading to substantial compression of the vessel from all sides after water absorption. Cell distribution also differed fundamentally between the two implantation strategies. A homogeneous cell distribution inside the matrix was adjusted for construct A, and cells were concentrated into a middle hydrogel layer for the design of group B.

Based on previous findings, where intrinsic vascularization could be demonstrated in ADA–GEL microcapsules (Steiner et al., 2018) and melanoma tumor growth in other alginate-based bioinks after a 4-week implantation time span in the AVL rat model (Schmid et al., 2024; Schmid et al., 2022), we used an equal implantation time span. We demonstrated that the shape of constructs had no effect on the bioink stability after implantation since the bioink constructs were easily removable from the isolation chambers and remained intact. Furthermore, these observations indicate that the implanted pre-cross-linked ADA–GEL is stable under *in vivo* conditions for 4 weeks in the AVL rat model. A newly formed mass of connective tissue was observed in the surrounding regions of the isolation chambers, primarily at the entrance of the chambers. In consistency with previous AVL rat model studies (Arkudas et al., 2013), our findings suggested that implanted hydrogel properties have no influence on the external environment; this again proved the isolating capacity of the chamber. The explanted structures, where subsequent tumor formation was identified, were partially or entirely darker in color (Figure 4). This discoloration can be caused by the macroscopically observed hemorrhages, which are a frequent component of the melanoma environment and have been observed in previous melanoma AVL explants (Schmid et al., 2024). In contrast to other alginate and gelatin-based hydrogels (alginate/hyaluronic acid/gelatin) and Matrigel AVL melanoma progression models (Schmid et al., 2024; Schmid et al., 2022), where a tumor growth was present, solid tumors did not grow out of the isolation chambers in the current ADA–GEL groups, independently of the scaffold shape.

Ideally, the scaffold degradation rate should be analogous to *de novo* tissue formation, allowing for the growing tissue to synchronously replace the degrading matrix (Rottensteiner et al., 2014). Adding mTG to the cross-linking solution, as performed in this study, revealed a slower degradation rate of ADA–GEL and can increase its applicability for long-term *in vivo* applications (Bider et al., 2024). In the present study, pre-cross-linked ADA–GEL allowed for fibrovascular tissue formation in the AVL rat model, without gaps between the matrix and tissue ingrowth, suggesting that the matrix did not degrade faster than the newly growing tissue replaced. After 4 weeks, *de novo* formed fibrovascular tissue growth was observed in pre-cross-linked ADA–GEL explants. However, a substantially higher rate of ingrown tissue was present in group B explants compared to group A, respectively (Figure 5). It must be kept in mind that group A’s AVLs were probably compressed due to the volumetric expansion of the hydrogel and suboptimal construct design; therefore, the blood flow was possibly obstructed. However, the fact that tissue formation was present and explanted constructs were partially perfused with yellow Microfil^®^ suggest that the AVLs of group A were temporarily patent.

Focusing on the histological evaluation of tumor formation, our study demonstrated that reducing the height of the bottom cylinder and omitting the outer wall of the implantation constructs positively influenced tumor formation when comparing groups A and B, presumably due to improved AVL patency. Although tumor formation in group A was barely present, quantification of tumor sizes of group B constructs resulted in a mean value of 42.46% (±31.47%) (Figure 6), showing similarity to other alginate-based, cast AVL melanoma progression models (Schmid et al., 2024). The tumor growth ability may also be additionally impacted by the cell distribution within the implantation scaffold. Because of the homogenous cell distribution within the scaffolds for group A, cells were detected to be located on the periphery of the matrix without direct contact to the AVL and were unable to survive until the neo-vessel development, which is known to occur between days 10 and 14 after implantation (Heltmann-Meyer et al., 2021) and predominantly emerges from the interposed venous graft (Weigand et al., 2018). Therefore, for the establishment of malignant tumor models, we recommend a compact cell distribution, as was used in the present study’s group B, where cells are concentrated in a middle layer, directly contacting the AVL from the first second of the implantation.

In cases when tumor growth was noted, we were able to characterize key features of tumor development in the tumor masses (Figure 7), which further confirms the AVL rat model’s applicability as a malignant melanoma model. Ki67 expression, as a marker of proliferation (Andrés-Sánchez et al., 2022), revealed that tumor cells were able to proliferate in the identified cancerous areas of the chamber explants. Furthermore, vimentin histology demonstrated the presence of mesenchymal cells both within and around the evolved tumor masses, strongly associating with the microenvironment characteristics of human melanoma (Hendrix et al., 1992). In primary malignant melanoma tumors, PAS-positive micro-capillaries are often reported as a developing intravasation that can support the metastasis formation (Schmid et al., 2022) and were also observed in the tumorous tissues of AVL chamber explants. Moreover, melanoma tumors are frequently characterized by an inhomogeneous SOX10 expression, as was observed in this study. SOX10 is a marker for melanoma plasticity, such as invasive tumor cell behavior. Reduced expression of SOX10 results in decreased proliferation and the emergence of invasive characteristics (Capparelli et al., 2022). One of the five animals in group A had a sizable tumor growth inside the AVL’s isolation chamber, whereas tumor growth areas also showed a great deal of variability in animals of group B, as did the newly formed fibrovascular tissue areas. The natural variability of the AVLs, combined with the unknown duration of AVL patency and, consequently, the length of time they were able to supply the tumor cells or the newly forming connective tissue, may help explain this phenomenon. At the same time, variations in the age and weight of the rats involved may potentially affect the results and lead to heterogeneity within one experimental group. Additionally, within-experimental-group heterogeneity is recognized as a potential issue in animal experiments (Voelkl and Würbel, 2024), although we maintained as many experimental parameters as feasible in a standardized manner, such as sex, period of implantation, or cage conditions.

Immune response was analyzed with regard to the presence of macrophages as inflammation markers based on anti-CD68 and anti-CD163 antibody stainings. The absence of multinucleated giant cells, along with the maintained wellbeing of the animals, indicates that good biocompatibility of pre-cross-linked ADA–GEL, consistent with previous AVL studies assessing the *in vivo* immune response to ADA–GEL compositions (Heltmann-Meyer et al., 2025). The accumulation pattern of macrophages and the average of presented macrophages per mm^2^ of histological cross sections did not differ significantly between the two different scaffold designs (Figure 8). This leads us to the conclusion that the shape of the implanted hydrogel has no impact on material biocompatibility. Likewise, the migration ability of macrophages within hydrogels seems to be related to the microporous structure characteristic of the material (Schmid et al., 2024; Dai et al., 2018). A formulation of ADA–GEL (2.5% w/v ADA–2.5% w/v GEL) displayed a range of inhomogeneous pore diameter between 0.02 µm and 1.6 µm (Detsch et al., 2015; Sprenger et al., 2024). Accordingly, ADA–GEL seems to be suited to promote monocytic immigration since macrophages can even migrate via pores of 0.22 µm (Stevens and Bähr, 1993). Furthermore, the presence of tumor-associated, anti-inflammatory, CD163-positive macrophages (Figure 9) suggests that the implanted melanoma cells can have an influence on the local immunological response, thereby creating a pro-cancerous, immunosuppressive, *in vivo*-like tumor environment (Falleni et al., 2017; Xu et al., 2025). This demonstrates the system’s biological significance for studying tumor-immune system crosstalk. Additionally, tumor-associated macrophages are known to stimulate tumor angiogenesis (Lamagna et al., 2006). Concordant with former studies implanting other ADA–GEL compositions (Heltmann-Meyer et al., 2025), we were able to demonstrate that the pre-cross-linked ADA–GEL promotes neovascularization within the implantation chamber (Figure 11), which is essential for nutrient and gas supply as well as for mimicking the tumor microenvironment the tumor microenvironment. The presence of newly formed vasculature in malignant tissue areas suggests activated tumor angiogenesis, which was likely induced by the implanted melanoma cells. As a result, angiogenesis, as an important hallmark of cancer (Majidpoor and Mortezaee, 2021), can be identified in our malignant melanoma model.

The presence of nodal metastasis of melanoma is crucial for clinical tumor stage classification and therapy. Cell migration and spreading capability of a melanoma cell line originating from a nodal metastasis, as used in the current study, embedded in pre-cross-linked ADA–GEL has been formerly described *in vitro* (Schipka et al., 2024). Interestingly, we were able to detect metastatic single melanoma cells or clusters in each lymph node explant (Supplementary Figure S2) of rats. As a result, tumor cells were able to migrate through the AVL vessels and cause metastasis, independently of scaffold morphology or the presence of primary tumor growth. In addition, animals from group B exhibited a higher degree of metastasis, which can be attributed to the improved scaffold implantation strategy, likely resulting in prolonged AVL patency and optimized cell distribution within the chamber (Figure 12). These findings show similarity to previous AVL melanoma progression models, where metastatic cells and clusters could be observed in explanted lungs and lymph nodes (Schmid et al., 2024), and underline the AVL model’s importance in tumor metastasis model development and its clinical relevance. Anastomosing the femoral vessels and a venous graft into a long-term patent AVL is the most crucial step of an AVL surgery. The procedure of an end-to-end anastomosis calls for microsurgical skills (Mengen et al., 2024), but there are further factors that must be considered during the subsequent surgical process. First, it is crucial that the AVL is transferred into the gel-filled chamber with minimal tension, ensuring it is neither kinked nor compressed along the vascular axis. This could mainly occur by the chamber walls or the plastic pins in the middle of the constructs used to prevent loop luxation. Second, the characteristics of the implanted material can also have an influence on the vessels and, therefore, can lead to a blocked blood flow. In contrast to cast hydrogel systems, used previously for AVL models, 3D-printed hydrogel grafts are pre-shaped and should be adapted precisely to the chambers and loop dimensions. To find a universally applicable 3D scaffold design due to the inherent variation across the animals and the diversity of the anastomosed AVL, regarding, for example, the vessel diameter or length, appeared to be more challenging than expected.

## Conclusion

As has already been demonstrated, the AVL small animal model is an outstanding method to overcome the limitations of non-vascularized *in vitro* cell cultivation techniques. It is also well suited to study various developmental stages of cancer and metastatic tumor behavior in a defined, precisely adjustable *in vivo* microenvironment. Moreover, it provides an opportunity to investigate the mechanisms underlying the development of drug resistance. However, well-standardized, 3D printing strategies still need to be properly established. The demonstrated differences between the scaffold shapes of groups A and B concerning fibrovascular tissue ingrowth and tumor formation can be explained by blood flow interruption due to swelling caused by material expansion and suboptimal design of the hydrogel-based biomaterial. Thus, we recommend assessing bioink characteristics, such as water intake capacity, and taking them into consideration when constructing 3D-printed hydrogel scaffolds for the confined space of the tissue container of the AVL model or similar studies.

## Data Availability

The original contributions presented in the study are included in the article/Supplementary Material; further inquiries can be directed to the corresponding author.

## References

[B1] Aazmi A. Zhang D. Mazzaglia C. Yu M. Wang Z. Yang H. (2024). Biofabrication methods for reconstructing extracellular matrix mimetics. Bioact. Mater 31, 475–496. 10.1016/j.bioactmat.2023.08.018 37719085 PMC10500422

[B2] Andrés-Sánchez N. Fisher D. Krasinska L. (2022). Physiological functions and roles in cancer of the proliferation marker Ki-67. J. Cell Sci. 135 (11), jcs258932. 10.1242/jcs.258932 35674256

[B3] Arkudas A. Balzer A. Buehrer G. Arnold I. Hoppe A. Detsch R. (2013). Evaluation of angiogenesis of bioactive glass in the arteriovenous loop model. Tissue Eng. Part C Methods 19 (6), 479–486. 10.1089/ten.tec.2012.0572 23189952 PMC3629783

[B4] Bider F. Miola M. Clejanu C. E. Götzelmann J. Kuth S. Vernè E. (2024). 3D bioprinting of multifunctional alginate dialdehyde (ADA)-gelatin (GEL) (ADA-GEL) hydrogels incorporating ferulic acid. Int. J. Biol. Macromol. 257 (Pt 2), 128449. 10.1016/j.ijbiomac.2023.128449 38029911

[B5] Capparelli C. Purwin T. J. Glasheen M. Caksa S. Tiago M. Wilski N. (2022). Targeting SOX10-deficient cells to reduce the dormant-invasive phenotype state in melanoma. Nat. Commun. 13 (1), 1381. 10.1038/s41467-022-28801-y 35296667 PMC8927161

[B6] Dai Y. Li X. Wu R. Jin Y. Gao C. (2018). Macrophages of different phenotypes influence the migration of BMSCs in PLGA scaffolds with different pore size. Biotechnol. J. 13 (1), 1700297. 10.1002/biot.201700297 28731632

[B7] Datta P. Ayan B. Ozbolat I. T. (2017). Bioprinting for vascular and vascularized tissue biofabrication. Acta Biomater. 51, 1–20. 10.1016/j.actbio.2017.01.035 28087487

[B8] Detsch R. Sarker B. Zehnder T. Frank G. Boccaccini A. R. (2015). Advanced alginate-based hydrogels. Mater. Today 18 (10), 590–591. 10.1016/j.mattod.2015.10.013

[B9] Dranseikiene D. Schrüfer S. Schubert D. W. Reakasame S. Boccaccini A. R. (2020). Cell-laden alginate dialdehyde-gelatin hydrogels formed in 3D printed sacrificial gel. J. Mater Sci. Mater Med. 31 (3), 31. 10.1007/s10856-020-06369-7 32152812 PMC7062650

[B10] Ege D. Boccaccini A. R. (2024). Investigating the effect of processing and material parameters of alginate dialdehyde-gelatin (ADA-GEL)-Based hydrogels on stiffness by XGB machine learning model. Bioeng. (Basel) 11 (5), 415. 10.3390/bioengineering11050415 38790283 PMC11117982

[B11] Falleni M. Savi F. Tosi D. Agape E. Cerri A. Moneghini L. (2017). M1 and M2 macrophages' clinicopathological significance in cutaneous melanoma. Melanoma Res. 27 (3), 200–210. 10.1097/cmr.0000000000000352 28272106

[B12] Feng W. Wang Z. (2023). Tailoring the swelling-shrinkable behavior of hydrogels for biomedical applications. Adv. Sci. 10 (28), 2303326. 10.1002/advs.202303326 37544909 PMC10558674

[B13] Fontoura J. C. Viezzer C. dos Santos F. G. Ligabue R. A. Weinlich R. Puga R. D. (2020). Comparison of 2D and 3D cell culture models for cell growth, gene expression and drug resistance. Mater Sci. Eng. C Mater Biol. Appl. 107, 110264. 10.1016/j.msec.2019.110264 31761183

[B14] Gungor-Ozkerim P. S. Inci I. Zhang Y. S. Khademhosseini A. Dokmeci M. R. (2018). Bioinks for 3D bioprinting: an overview. Biomater. Sci. 6 (5), 915–946. 10.1039/c7bm00765e 29492503 PMC6439477

[B15] Harries M. Malvehy J. Lebbe C. Heron L. Amelio J. Szabo Z. (2016). Treatment patterns of advanced malignant melanoma (stage III-IV) - a review of current standards in Europe. Eur. J. Cancer 60, 179–189. 10.1016/j.ejca.2016.01.011 27118416

[B16] Hazur J. Detsch R. Karakaya E. Kaschta J. Teßmar J. Schneidereit D. (2020). Improving alginate printability for biofabrication: establishment of a universal and homogeneous pre-crosslinking technique. Biofabrication 12 (4), 045004. 10.1088/1758-5090/ab98e5 32485692

[B17] Heltmann-Meyer S. Steiner D. Müller C. Schneidereit D. Friedrich O. Salehi S. (2021). Gelatin methacryloyl is a slow degrading material allowing vascularization and long-term usein vivo. Biomed. Mater 16 (6), 065004. 10.1088/1748-605x/ac1e9d 34406979

[B18] Heltmann-Meyer S. Detsch R. Hazur J. Kling L. Pechmann S. Kolan R. R. (2025). Biofunctionalization of ADA-GEL hydrogels based on the degree of cross-linking and polymer concentration improves angiogenesis. Adv. Healthc. Mater 14 (11), e2500730. 10.1002/adhm.202500730 40095294 PMC12023838

[B19] Hendrix M. J. Seftor E. A. Chu Y. W. Seftor R. E. B. Nagle R. B. McDaniel K. M. (1992). Coexpression of vimentin and keratins by human melanoma tumor cells: correlation with invasive and metastatic potential. J. Natl. Cancer Inst. 84 (3), 165–174. 10.1093/jnci/84.3.165 1371813

[B20] Jain N. Kejariwal M. Chowdhury F. I. Noor I. Savilov S. Yahya M. (2024). Synthesis, characterization and application of hydrogel for cancer treatment. Chem. Phys. Impact 9, 100737. 10.1016/j.chphi.2024.100737

[B21] Jenkins R. W. Fisher D. E. (2021). Treatment of advanced melanoma in 2020 and beyond. J. Invest Dermatol 141 (1), 23–31. 10.1016/j.jid.2020.03.943 32268150 PMC7541692

[B22] Khan M. U. A. Aslam M. A. Abdullah M. F. B. Al-Arjan W. S. Stojanovic G. M. Hasan A. (2024). Hydrogels: classifications, fundamental properties, applications, and scopes in recent advances in tissue engineering and regenerative medicine – a comprehensive review. Arabian J. Chem. 17, 105968. 10.1016/j.arabjc.2024.105968

[B23] Lamagna C. Aurrand-Lions M. Imhof B. A. (2006). Dual role of macrophages in tumor growth and angiogenesis. J. Leukoc. Biol. 80 (4), 705–713. 10.1189/jlb.1105656 16864600

[B24] Landau S. Okhovatian S. Zhao Y. Liu C. Shakeri A. Wang Y. (2024). Bioengineering vascularization. Development 151 (23), dev204455. 10.1242/dev.204455 39611864 PMC11698057

[B25] Lugano R. Ramachandran M. Dimberg A. (2020). Tumor angiogenesis: causes, consequences, challenges and opportunities. Cell Mol. Life Sci. 77 (9), 1745–1770. 10.1007/s00018-019-03351-7 31690961 PMC7190605

[B26] Majidpoor J. Mortezaee K. (2021). Angiogenesis as a hallmark of solid tumors - clinical perspectives. Cell Oncol. (Dordr) 44 (4), 715–737. 10.1007/s13402-021-00602-3 33835425 PMC12980750

[B27] Malandrino A. Zhang H. Schwarm N. Böhringer D. Kah D. Kuster C. (2024). Plasticity of 3D hydrogels predicts cell biological behavior. Biomacromolecules 25 (12), 7608–7618. 10.1021/acs.biomac.4c00765 39512191 PMC11632650

[B28] Mengen L. M. Horch R. E. Arkudas A. (2024). Mikrochirurgische gefäßnaht. Oper. Orthop. Traumatol. 36 (6), 307–319. 10.1007/s00064-024-00869-3 39537936

[B29] Monavari M. Homaeigohar S. Fuentes-Chandía M. Nawaz Q. Monavari M. Venkatraman A. (2021). 3D printing of alginate dialdehyde-gelatin (ADA-GEL) hydrogels incorporating phytotherapeutic icariin loaded mesoporous SiO(2)-CaO nanoparticles for bone tissue engineering. Mater Sci. Eng. C Mater Biol. Appl. 131, 112470. 10.1016/j.msec.2021.112470 34857258

[B30] Osterloh J. Heltmann-Meyer S. Trossmann V. T. Cai A. Kulicke Y. Terörde K. (2025). *In vivo* vascularization of cell-supplemented spider silk-based hydrogels in the arteriovenous loop model. Biomimetics (Basel) 10 (2), 117. 10.3390/biomimetics10020117 39997140 PMC11853081

[B31] Parrish J. Lim K. Zhang B. Radisic M. Woodfield T. B. (2019). New frontiers for biofabrication and bioreactor design in microphysiological system development. Trends Biotechnol. 37 (12), 1327–1343. 10.1016/j.tibtech.2019.04.009 31202544 PMC6874730

[B32] Paulsen S. J. Miller J. S. (2015). Tissue vascularization through 3D printing: will technology bring us flow? Dev. Dyn. 244 (5), 629–640. 10.1002/dvdy.24254 25613150

[B33] Polykandriotis E. Arkudas A. Beier J. P. Hess A. Greil P. Papadopoulos T. (2007). Intrinsic axial vascularization of an osteoconductive bone matrix by means of an arteriovenous vascular bundle. Plast. Reconstr. Surg. 120 (4), 855–868. 10.1097/01.prs.0000277664.89467.14 17805112

[B34] Rebecca V. W. R. (2020). Pre-clinical modeling of cutaneous melanoma. Nat. Commun. 11 (1), 2858. 10.1038/s41467-020-15546-9 32504051 PMC7275051

[B35] Reakasame S. Boccaccini A. R. (2018). Oxidized alginate-based hydrogels for tissue engineering applications: a review. Biomacromolecules 19 (1), 3–21. 10.1021/acs.biomac.7b01331 29172448

[B36] Rottensteiner U. Sarker B. Heusinger D. Dafinova D. Rath S. Beier J. (2014). *In vitro* and *in vivo* biocompatibility of alginate dialdehyde/gelatin hydrogels with and without nanoscaled bioactive glass for bone tissue engineering applications. Mater. (Basel) 7 (3), 1957–1974. 10.3390/ma7031957 28788549 PMC5453292

[B37] Ruther F. Distler T. Boccaccini A. R. Detsch R. (2018). Biofabrication of vessel-like structures with alginate di-aldehyde-gelatin (ADA-GEL) bioink. J. Mater Sci. Mater Med. 30 (1), 8. 10.1007/s10856-018-6205-7 30594988

[B38] Santoni S. Gugliandolo S. G. Sponchioni M. Moscatelli D. Colosimo B. M. (2022). 3D bioprinting: current status and trends—a guide to the literature and industrial practice. Bio-Design Manuf. 5 (1), 14–42. 10.1007/s42242-021-00165-0

[B39] Sarker B. Papageorgiou D. G. Silva R. Zehnder T. Gul-E-Noor F. Bertmer M. (2014a). Fabrication of alginate–gelatin crosslinked hydrogel microcapsules and evaluation of the microstructure and physico-chemical properties. J. Mater. Chem. B 2 (11), 1470–1482. 10.1039/c3tb21509a 32261366

[B40] Sarker B. Singh R. Silva R. Roether J. A. Kaschta J. Detsch R. (2014b). Evaluation of fibroblasts adhesion and proliferation on alginate-gelatin crosslinked hydrogel. PLoS One 9 (9), e107952. 10.1371/journal.pone.0107952 25268892 PMC4182442

[B58] Schindelin J. Arganda-Carreras I. Frise E. Kaynig V. Longair M. Pietzsch T. (2012). Fiji: an open-source platform for biological-image analysis. Nature Methods 9 (7), 676 – 682. 10.1038/nmeth.2019 22743772 PMC3855844

[B41] Schipka R. Heltmann-Meyer S. Schneidereit D. Friedrich O. Röder J. Boccaccini A. R. (2024). Characterization of two different alginate-based bioinks and the influence of melanoma growth within. Sci. Rep. 14 (1), 12945. 10.1038/s41598-024-63642-3 38839791 PMC11153560

[B42] Schmid R. Schmidt S. K. Hazur J. Detsch R. Maurer E. Boccaccini A. R. (2020). Comparison of hydrogels for the development of well-defined 3D cancer models of breast cancer and melanoma. Cancers 12 (8), 2320. 10.3390/cancers12082320 32824576 PMC7465483

[B43] Schmid R. Schmidt S. K. Detsch R. Horder H. Blunk T. Schrüfer S. (2022). A new printable alginate/hyaluronic acid/gelatin hydrogel suitable for biofabrication of *in vitro* and *in vivo* metastatic melanoma models. Adv. Funct. Mater. 32 (2), 2107993. 10.1002/adfm.202107993

[B44] Schmid R. Schmidt S. K. Schrüfer S. Schubert D. W. Heltmann-Meyer S. Schicht M. (2024). A vascularized *in vivo* melanoma model suitable for metastasis research of different tumor stages using fundamentally different bioinks. Mater. Today Bio 26, 101071. 10.1016/j.mtbio.2024.101071 38736612 PMC11081803

[B45] Schulik J. Salehi S. Boccaccini A. Schrüfer S. Schubert D. Arkudas A. (2023). Comparison of the behavior of 3D-Printed endothelial cells in different bioinks. Bioeng. (Basel) 10 (7), 751. 10.3390/bioengineering10070751 37508778 PMC10376299

[B46] Shukla P. Yeleswarapu S. Heinrich M. A. Prakash J. Pati F. (2022). Mimicking tumor microenvironment by 3D bioprinting: 3D cancer modeling. Biofabrication 14 (3), 032002. 10.1088/1758-5090/ac6d11 35512666

[B47] Singvogel K. Schittek B. (2024). Dormancy of cutaneous melanoma. Cancer Cell Int. 24 (1), 88. 10.1186/s12935-024-03278-5 38419052 PMC10903048

[B48] Sprenger L. Lu H. H. Trippmacher S. Mansfeld U. Milkin P. Ionov L. (2024). Composite alginate dialdehyde-gelatin (ADA-GEL) hydrogel containing short ribbon-shaped fillers for skeletal muscle tissue biofabrication. ACS Appl. Mater Interfaces 16 (34), 44605–44622. 10.1021/acsami.4c10751 39159061

[B49] Steiner D. Lingens L. Fischer L. Köhn K. Detsch R. Boccaccini A. R. (2018). Encapsulation of mesenchymal stem cells improves vascularization of alginate-based scaffolds. Tissue Eng. Part A 24 (17-18), 1320–1331. 10.1089/ten.tea.2017.0496 29652607

[B50] Stevens A. Bähr M. (1993). Origin of macrophages in central nervous tissue. A study using intraperitoneal transplants contained in millipore diffusion chambers. J. Neurol. Sci. 118 (2), 117–122. 10.1016/0022-510x(93)90100-d 8229059

[B51] Tas F. (2012). Metastatic behavior in melanoma: timing, pattern, survival, and influencing factors. J. Oncol. 2012, 647684. 10.1155/2012/647684 22792102 PMC3391929

[B52] Tur C. Eckstein M. Bucci L. Auth J. Bergmann C. Rauber S. (2025). Effects of different B-cell-depleting strategies on the lymphatic tissue. Ann. Rheumatic Dis. 10.1016/j.ard.2025.06.2120 40651933

[B53] Voelkl B. Würbel H. (2024). Heterogeneity of animal experiments and how to deal with it. Lab. Anim. 58 (5), 493–497. 10.1177/00236772241260173 39315551

[B54] Weigand A. Beier J. P. Arkudas A. Al-Abboodi M. Polykandriotis E. Horch R. E. (2016). The arteriovenous (AV) loop in a small animal model to study angiogenesis and vascularized tissue engineering. J. Vis. Exp. 117. 10.3791/54676 27842348 PMC5226146

[B55] Weigand A. Horch R. Boos A. Beier J. Arkudas A. (2018). The arteriovenous loop: engineering of axially vascularized tissue. Eur. Surg. Res. 59 (3-4), 286–299. 10.1159/000492417 30244238

[B56] Xu J. Ding L. Mei J. Hu Y. Kong X. Dai S. (2025). Dual roles and therapeutic targeting of tumor-associated macrophages in tumor microenvironments. Signal Transduct. Target. Ther. 10 (1), 268. 10.1038/s41392-025-02325-5 40850976 PMC12375796

[B57] Zhan W. Marre D. Mitchell G. M. Morrison W. A. Lim S. Y. (2016). Tissue engineering by intrinsic vascularization in an *in vivo* tissue engineering chamber. J. Vis. Exp. (111), 54099. 10.3791/54099 27286267 PMC4927750

