# Supplementary material for: Biofabrication of 3D-printed, pre-cross-linked alginate dialdehyde–gelatin (ADA–GEL) scaffolds for an in vivo metastatic arteriovenous loop tumor model
Source: Front Bioeng Biotechnol. 2025 Nov 3;13:1657653. doi: 10.3389/fbioe.2025.1657653 (PMC12620393; doi:10.3389/fbioe.2025.1657653)
Supplement: Supplementary file 1 [file DataSheet1.docx]

Supplementary material


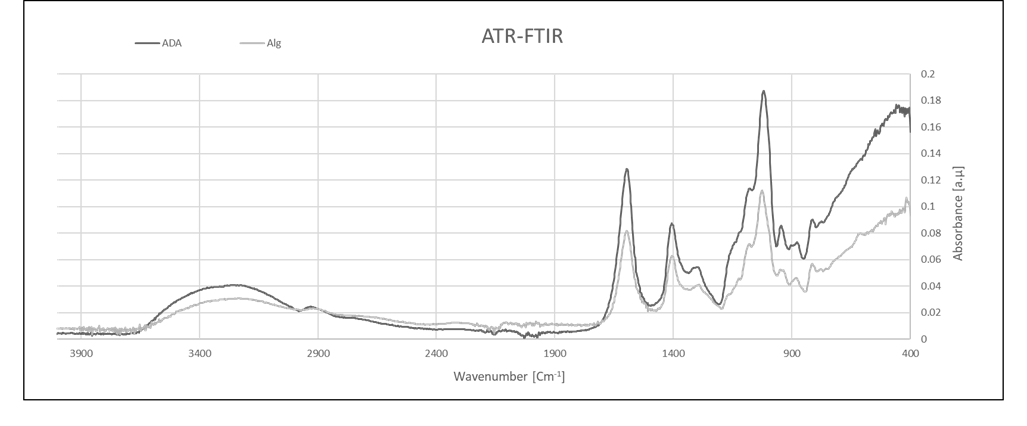


Supl.Fig.1. ATR-FTIR spectroscopy of synthetized ADA and pure alginate. Both ADA and alginate showed broad absorption bands in the range of 3,200-3,500 cm⁻¹, corresponding to O-H stretching vibrations of hydroxyl groups, which shows in polysaccharide-based materials. In alginate a highlight peak around 1,600–1,650 cm⁻¹ was observed, corresponding to asymmetric stretching of carboxylate groups (COO⁻), whereas ADA showed a relatively stronger signal in the same region, indicating a higher content of carboxyl functional groups. Peaks in the 1,000–1,100 cm⁻¹ region were assigned to C–O–C and C–O stretching vibrations, confirming the saccharide backbone structure. C-C stretching vibration peak was shown in 1,300-1,400 cm⁻¹.


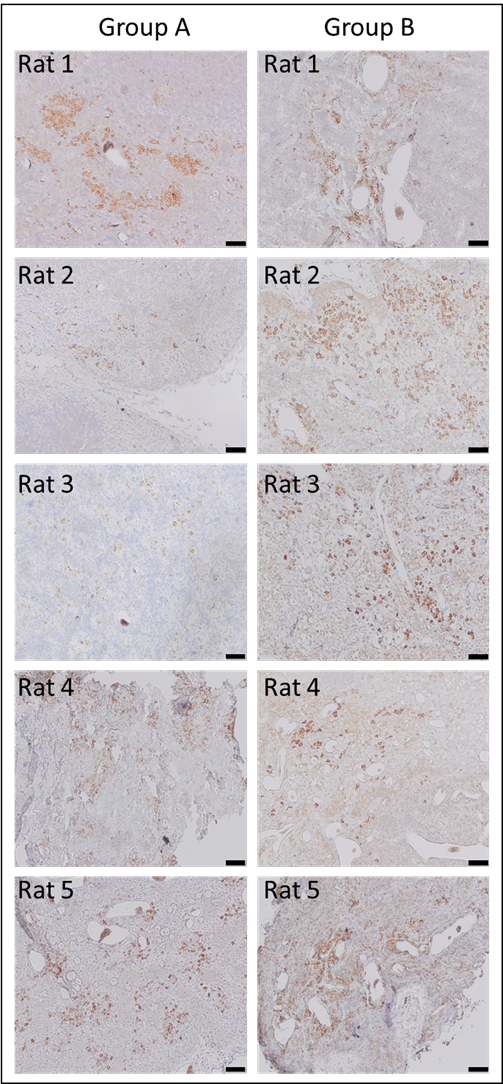


Supl.Fig.2. HMB45 stained histological cross sections of each animal lymph nodes explants, demonstrating metastatic melanoma cells. HMB45-positive cells are colored in brown. scale bars = 50 µm.
